# Semi-metric optimal transport enables robust spatial multi-omics integration

**DOI:** 10.21203/rs.3.rs-10505162/v1

**Published:** 2026-09-18

**Authors:** Shuang Wang, Yikun Bai, Ricardo Melo Ferreira, Huy Tran, Hengrong Du, Xuhong Zhang, Ying-Hua Cheng, Angela R. Sabo, Sanjay Jain, Pierre C. Dagher, Tarek M. El-Achkar, Michael T Eadon, Soheil Kolouri, Juexin Wang

**Affiliations:** 1. Department of Computer Science, Indiana University Bloomington, Bloomington, IN, USA; 2. Department of Mathematics, Purdue University, West Lafayette, IN, USA; 3. Department of Medicine, Indiana University School of Medicine, Indianapolis, IN, USA; 4. Department of Computer Science, Vanderbilt University, Nashville, TN, USA; 5. Department of Mathematics and Computer Science, Fisk University, Nashville, TN, USA; 6. Division of Nephrology, Department of Medicine and Kidney Translational Research Center, Washington University School of Medicine, St. Louis, MO 63110, USA; 7. Department of Biomedical Engineering and Informatics, Indiana University Indianapolis, Indianapolis, IN, USA

## Abstract

Integrating heterogeneous spatial multi-omics data to uncover underlying biological and pathological mechanisms remains a major challenge, while existing approaches generally lack mathematically justified distances for comparing complex spatial tissues. We introduce **Spa**tial **O**ptimal **T**ransport (SpaOT), a spatial multi-omics integration framework based on a theoretically grounded semi-metric formulation of unbalanced optimal transport. Utilizing a total-variation-relaxed Fused Partial Gromov-Wasserstein strategy, SpaOT establishes a mathematically consistent distance geometry that enables stable biological comparison, representative tissue barycenters, and robust downstream analyses while accommodating differences in cellular composition, measurement technologies, spatial resolution, and noise. Across multiple studies with diverse spatial omics, SpaOT enables robust and accurate alignment, integration, and stratifications across samples, resolutions, and omics modalities in complex disease-related biological systems. These capabilities facilitate the identification of biologically meaningful cellular organization, molecular signatures, and spatial relationships, establishing SpaOT as a general foundation for spatial multi-omics integration and comparative analysis.

Spatial omics technologies^1^ are transforming the study of biological systems by enabling spatially resolved measurement of complementary molecular modalities, including gene expression^2^, protein abundance^3^, and metabolite concentrations^4^, within their native tissue context. A central data analysis challenge is heterogeneous spatial omics integration, which is complicated by batch effects from biological variability across samples^5^, technological disparities in measurement platforms^6,7^, and heterogeneity across omics modalities^8^.

Existing spatial omics integration can be categorized into representation alignment and Optimal Transport (OT)-based strategies. Representation alignment methods learn representations in a shared space, including SpatialGlue^9^, aligning different modalities into a shared embedding, MISO^10^, fusing multi-modal spatial omics by concatenation and filtration, and INSPIRE^11^, aligning non-negative matrix factorization. However, existing representation methods may distort certain geometric relations in dimensional reduction^12,13^. In contrast, OT^14,15^ learns an explicit transport plan detailing which specific parts of the source transport to specific locations in the target. For example, moscot^16^ and PASTE2^17^ often jointly optimize feature similarity (e.g., gene expression distances) and geometric structure (e.g., spatial distances). These OT-based methods are naturally interpretable and usually achieve better generalizability^15^ without incurring information loss in representation learning^14^. Although 3d-OT^18^ and Graspot^19^ combine representation with OT, unbalanced OT^16,20^ typically relies on Kullback-Leibler (KL) divergence to accommodate mass variation between source and target distributions. Their consequent results are not supported by rigorous metric or semi-metric guarantees^21^, limiting consistent biological comparison across heterogeneous datasets.

We present SpaOT (**Spa**tial **O**ptimal **T**ransport), a spatial multi-omics integration framework based on Fused Partial Gromov-Wasserstein (FPGW)^22^ distance with theoretically proven semi-metric properties. By relaxing the requirement of equal total mass between datasets through a total variation penalty, SpaOT provides a flexible formulation that is robust to unequal sample mass, missing information, and noise. To the best of our knowledge, it is the first theoretically grounded semi-metric formulation of unbalanced OT that enables mathematically consistent distance measurement in spatial omics research. In colorectal carcinoma (CRC) and translational breast cancer studies, SpaOT enables disease stratification utilizing within-modality alignment. For cross-modality integration, it outperformed competitive methods in mouse cerebellum and multi-resolution mapping in CRC studies. Furthermore, in chronic kidney disease (CKD) research, SpaOT-enabled integration of complementary Xenium and Visium HD profiles revealed biologically meaningful pathways and ligand-receptor interactions within glomerular regions, providing deeper insights into disease-associated spatial cellular organization.

## RESULTS

### Overview of SpaOT

**Figure 1 ∣:**
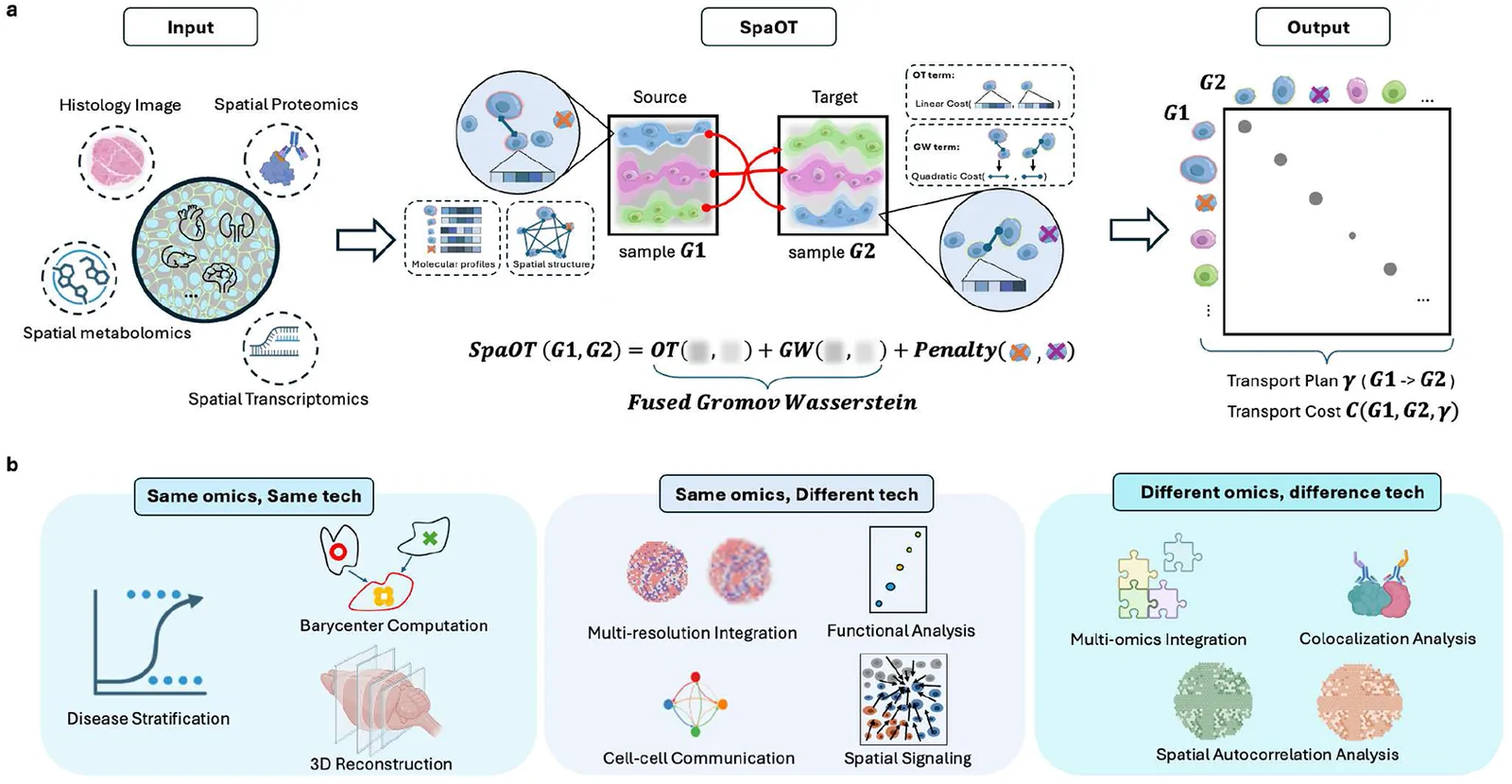
Overview of SpaOT. **a,** Schematic illustration of the SpaOT framework. SpaOT integrates diverse spatial data types, including spatial transcriptomics, spatial proteomics, spatial metabolomics, and histology imaging. SpaOT formulates cross-sample correspondence from source sample G1 to target sample G2 using a fused Gromov-Wasserstein optimal transport strategy with additional penalty constraints (cross-labeled) to leverage molecular profiles (the OT term) and structural information (the GW term). The method estimates a transport plan γ between G1 and G2 and a transport cost C (Eq. (8) in Methods). **b,** Representative downstream applications in spatial omics integration.

Given a pair of spatial omics samples, SpaOT formulates the integration as an FPGW task to map the source to the target, enabling heterogeneous data alignment with unequal mass. Across diverse omics modalities, including spatial transcriptomics, spatial metabolomics, spatial proteomics, and histology imaging, each cell or spot is represented as a unit, while molecular profiles are treated as feature vectors (the OT term). Spatial distances among units define the underlying geometry of each sample, providing the pairwise structural costs (the GW term). The goal of SpaOT is to minimize the transport cost by jointly leveraging molecular profiles and structural information in a metric-measure (mm) space to obtain the transport plan mapping from source to target (Fig. 1a). We prove that SpaOT defines a semi-metric on metric-measure spaces (Proof in **Supplementary Notes C, D**), providing a theoretically grounded discrepancy measure for partial alignment (Ablation analysis in **Supplementary Fig. 1**).

The resulting transport cost and transport plan can be applied to a wide range of downstream tasks in spatial omics research. The transport cost can be treated as a metric to measure discrepancies between spots in mm-space, which enables consistent distance geometry and stable biological comparison in sample-level disease stratification. This metric can be averaged to describe the characteristics shared by samples in the same category as a barycenter. The transport plan enables the reconstruction of 3D tissue architecture by stacking the serial 2D sections and supports multi-resolution integration, providing insights into tissue function and spatial cell-cell interactions. Furthermore, it facilitates cross-omics integration, obtaining a comprehensive understanding of biological systems from multiple molecular perspectives (Fig. 1b).

### SpaOT is robust to different types of noise in simulations

**Figure 2 ∣:**
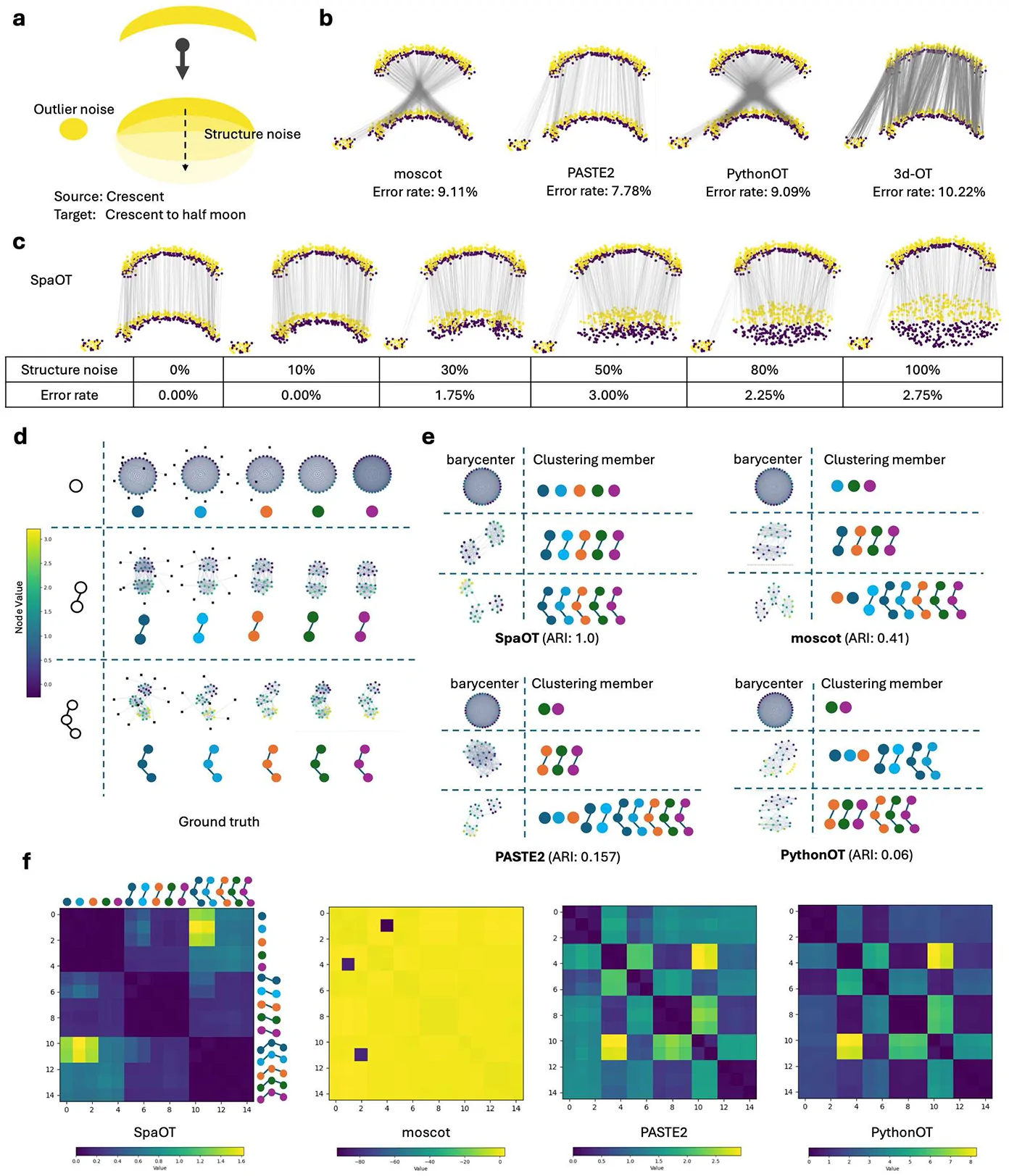
Simulation studies on object mapping and graph clustering using SpaOT. **a,** Schematic illustration of the simulated noise settings for benchmarking. Outlier noise was introduced by adding scattered points outside the target, whereas structural noise was generated by continuously transforming the target geometry from a crescent to a full-moon structure. **b,** Error rate comparison with representative OT-based methods under outlier noise, which was bounded by 9.09% (10%/110%). **c,** Mapping accuracy with increasing levels of structural noise in point clouds. The target point cloud was progressively interpolated from crescent-shaped to a perturbed full moon while maintaining fixed outlier contamination. Grey lines indicate transport correspondences between the source and target. **d,** Synthetic graph datasets used for clustering evaluation. Three graph categories with different community numbers (1-3), each containing five graph instances with varying levels of outlier-node contamination. The color of nodes denotes the value of node features (yellow to purple), and black nodes indicate outliers. To simplify the membership relations, each synthetic graph instance is represented by a solid ball, e.g., synthetic graphs with three communities are presented by three connected balls. **e,** Graph clustering results based on OT-derived graph distances and barycenter estimation. For results from each method, the first columns are k-means centroids of each cluster generated by barycenter estimation, while the subsequent columns display the graphs assigned to each centroid visualized by the simplified balls corresponding to the membership in **d**. Clustering accuracy was evaluated using the Adjusted Rand Index (ARI), which measures the agreement between the identified and true cluster assignments. **f,** Heatmap of pairwise graph distance matrices computed using different OT-based methods. Distance values correspond to transport costs derived from each method. All 15 synthetic graph instances from three categories were included in the computation of the distance matrices.

Beyond theoretical analysis, we evaluated the robustness enabled by semi-metric properties using synthetic datasets mapping between crescent-shaped point clouds. We introduced two types of noise: (i) outlier noise, where an additional 10% of scattered data points were added outside the target to distract the mapping process, and (ii) structural noise, in which the target geometry was progressively deformed from a crescent into a perturbed full moon shape (Fig. 2a). We compared SpaOT with several representative OT-based methods, including moscot^16^, PASTE2^17^, PythonOT^23,24^, and 3d-OT^18^.

SpaOT consistently avoided mapping source points to outliers, demonstrating robustness compared to erroneous mappings in competitive methods even in the absence of structural perturbation (Fig. 2b). As structural noise increased and the target geometry gradually transformed from a crescent (0%) to a full moon (100%), SpaOT maintained low error rates (Fig. 2c).

We further designed graph clustering tasks to measure the metric properties of OT-based approaches. The synthetic dataset comprised three graph categories containing one, two, and three communities, respectively. Each category consisted of five independently generated graph instances with random outlier nodes (Fig. 2d). Graphs were clustered using the K-means algorithm (K = 3), where pairwise distances were defined by the transport costs computed by SpaOT, moscot, PASTE2, and PythonOT. Cluster centroids were represented by barycenters^25^ that summarize the shared structural and feature characteristics of graphs within each cluster.

SpaOT correctly recognized all three graph categories and recovered their corresponding barycenters, whereas competitive methods were sensitive to outliers (Fig. 2e). Its pairwise distance matrices further exhibited clear within-category similarity and between-category separation, demonstrating a discriminative measure of graph similarity capturing intrinsic community structure (Fig. 2f).

### SpaOT captures consistent distance geometry and builds prototypes of disease-associated spatial organization in colorectal cancer

**Figure 3 ∣:**
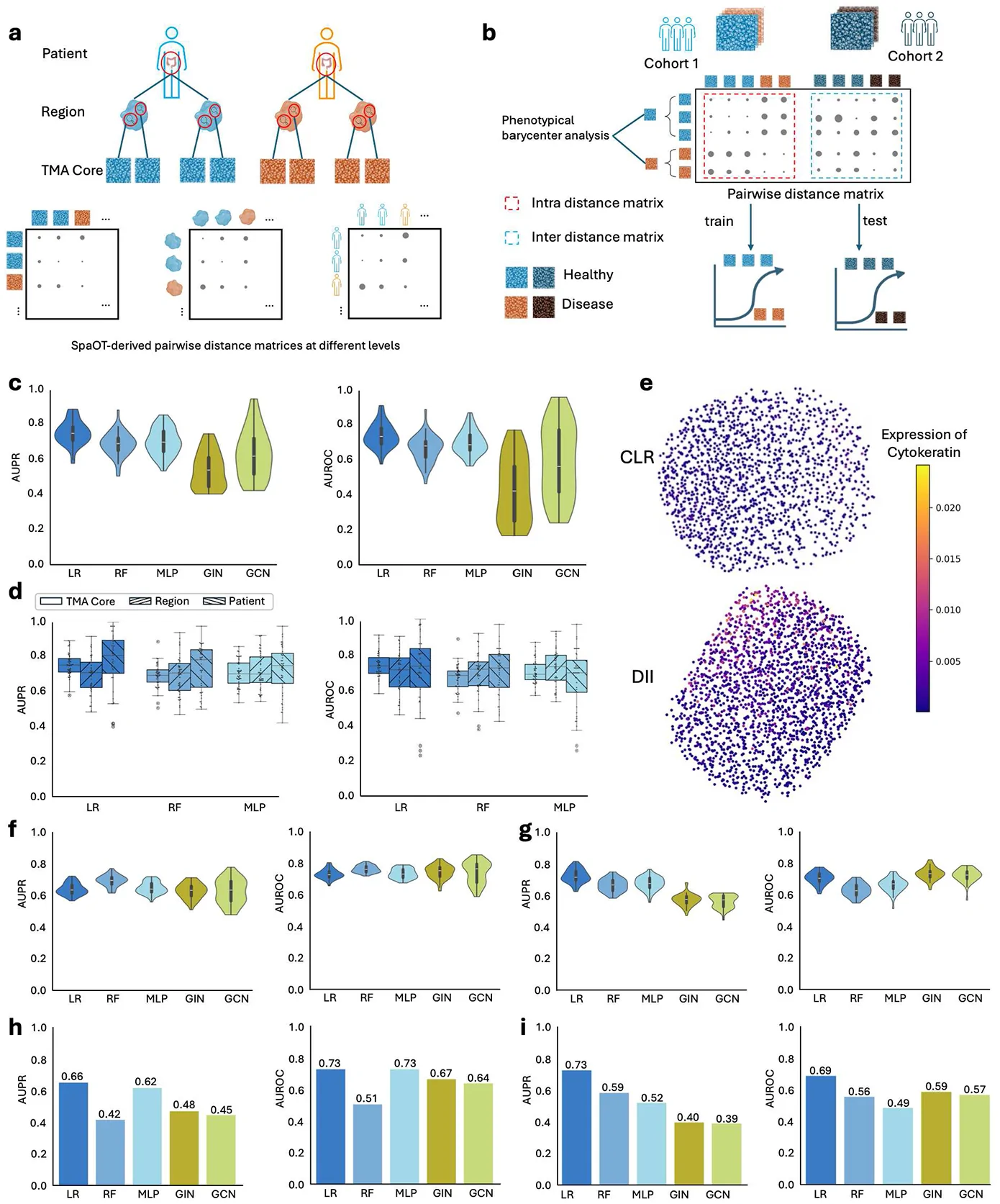
Benchmark SpaOT disease stratification for spatial proteomics samples from intra-cohort and inter-cohort sources. **a,** Hierarchical organization of the CRC spatial proteomics dataset. Multiple tissue regions were sampled from the same patient, and multiple TMA cores may originate from the same region, enabling computation of FPGW barycenters at both region and patient levels. **b,** Disease stratification workflow using SpaOT-derived pairwise distances. Spatial proteomics samples from different cohorts were represented as mm spaces integrating protein profiles and cellular spatial coordinates. Pairwise SpaOT distances were computed to generate intra- and inter-condition distance matrices, which were subsequently used for downstream stratification using machine learning models. Barycenter analysis can also be applied to both healthy and disease groups. **c,** Performance of CRC immune subtype stratification at the TMA core level (n = 140) using SpaOT-derived distance matrices and LR, RF, MLP, GIN, and GCN classifiers. Results are reported as AUPR (left) and AUROC (right). **d,** Comparison of stratification performance across different hierarchical representations, including TMA core-level, and region-level and patient-level barycentric aggregation using SpaOT-derived distance matrices and LR, RF, and MLP classifiers. **e,** Visualization of SpaOT barycenters for the two CRC immune subtypes, i.e., Crohn’s-like reaction (CLR) and diffuse immune infiltrate (DII), using cytokeratin expression as an example marker. Intra-dataset breast cancer subtype stratification results using spatial proteomics datasets from the Jackson cohort (**f**) and METABRIC cohort (**g**). Cross-dataset breast cancer subtype stratification evaluating robustness to cohort-specific biases, where (**h**) models are trained on METABRIC and tested on Jackson, and (**i**) model trained on Jackson and tested on METABRIC. CRC: colorectal cancer, LR: Logistic Regression, RF: Random Forest, MLP: Multilayer Perceptron, GIN: Graph Isomorphism Network, GCN: Graph Convolutional Network, AUPR: Area Under the Precision-Recall Curve, AUROC: Area Under the Receiver Operating Characteristic.

Next, we investigated whether SpaOT-derived transport cost can be treated as distances capturing differences between disease conditions in colorectal cancer (CRC)^26^. The explored dataset contains 140 Tissue Microarray (TMA) cores from 70 regions of 35 patients, representing two immune subtypes: Crohn’s-Like Reaction (CLR; 17 patients, 34 regions, 68 TMA cores) and Diffuse Inflammatory Infiltration (DII; 18 patients, 36 regions, 72 TMA cores), profiled using CO-Detection by indEXing (CODEX) (Fig. 3a).

For each sample, we computed SpaOT-derived cost as pairwise distances using spatial coordinates and protein expression profiles of all detected cells. This distance matrix was used as input to lightweight machine learning (ML) classifiers for disease subtype stratification, including logistic regression (LR), Random Forest (RF), and Multilayer Perceptron (MLP) (Fig. 3b). We compared SpaOT with widely adopted Graph neural network (GNN)-based approaches^27^, including Graph Isomorphism Network (GIN)^28^ and Graph Convolutional Network (GCN)^29^. Performance was assessed using five times 3-fold cross-validation with the Area Under the Precision-Recall Curve (AUPR) and the Area Under the Receiver Operating Characteristic Curve (AUROC).

At the TMA core level (n=140), SpaOT substantially outperformed GNN-based methods across all classifiers (Fig. 3c). Using LR, SpaOT outperformed GIN and GCN. Similar improvements were observed using RF and MLP. When evaluated at the region (n = 70) and patient (n = 35) levels (Fig. 3d), patient-level barycenters achieved the highest performance with LR, outperforming both the region-level representations and the TMA core-level. Consistent trends were observed for RF and MLP, indicating that SpaOT barycenters effectively summarize disease-associated spatial organization across multiple biological scales.

To further characterize subtype-specific spatial patterns, we computed barycenters as prototypes for CLR and DII samples and visualized the expression of cytokeratin, a tumor-cell marker (Fig. 3e). The CLR barycenter showed lower cytokeratin expression and stronger spatial heterogeneity, consistent with its organized immune architecture and enhanced anti-tumor response. In contrast, the DII barycenter displayed higher cytokeratin expression concentrated along the tissue boundary and a more homogeneous cellular organization.

### SpaOT exhibits generalizability and tolerance to site-specific biases in translational breast cancer stratification

We next evaluated the generalizability of SpaOT-derived distances across independent breast cancer datasets of the METABRIC cohort^30^ and the Jackson cohort^31^. Both datasets represent distinct patient populations profiled using the same technology Imaging Mass Cytometry (IMC).

In intra-cohort stratification, where ML models were trained and tested within the same dataset (Fig. 3f), SpaOT consistently outperformed GNN-based methods. On the Jackson dataset, SpaOT combined with RF classifier exceeded the performance of GIN and GCN. Similar results were observed for the METABRIC cohort (Fig. 3g).

Cross-cohort settings apply models trained on one cohort directly to the other, which is challenging due to severe batch effects from site-specific biases^7^. SpaOT demonstrated strong predictive performance and transferability across cohorts, while GNN-based methods exhibited limited generalization and remain impractical. When trained on METABRIC and tested on Jackson, SpaOT with LR classifier achieved an AUPR of 0.66 and an AUROC of 0.73 (Fig. 3h). Conversely, training on Jackson and testing on METABRIC achieved an AUPR of 0.73 and an AUROC of 0.69 (Fig. 3i). In comparison, GIN achieved remarkably lower performance in the corresponding settings (AUPR/AUROC: 0.48/0.67 and 0.40/0.59 , respectively), while GCN showed similar results in limited cross-cohort transferability.

### SpaOT faithfully aligns 2D tissue sections in 3D reconstruction

Reconstructing 3D tissue architecture by aligning consecutive adjacent 2D tissue sections has emerged as a powerful approach for studying tissue organization. We evaluated SpaOT using affine (rigid) transformations derived from OT transport plans on a dataset of 40 coronal half-mouse brain slices spanning the entire anterior-posterior axis^32,33^. SpaOT consistently outperformed competitive methods in nearest-neighbor label accuracy, centroid distance, and expression cosine similarity (**Supplementary Fig. 2a, b**). The resulting 3D reconstruction faithfully preserved the global spatial organization of major brain regions annotated by the Allen Brain Atlas^34^ (**Supplementary Fig. 2c**). Key anatomical structures, including the isocortex, striatum, and thalamus, were reconstructed in positions consistent with their known neuroanatomical locations and marker gene expression patterns. Specifically, *Rasgrf2* expression localized to the exterior cortical regions, *Gpr88* was enriched more anteriorly toward the olfactory bulb region, and *Rora* was concentrated in posterior regions toward the cerebellum, recapitulating established spatial organization.

We further evaluated SpaOT on a MERFISH dataset comprising 12 mouse hypothalamic preoptic area slices with known z-axis ordering^32,35^. SpaOT produced substantially more accurate adjacent-slice alignments than competing methods. (**Supplementary Fig. 3**).

### SpaOT accurately integrates spatial transcriptomics across multi-resolution platforms

**Figure 4 ∣:**
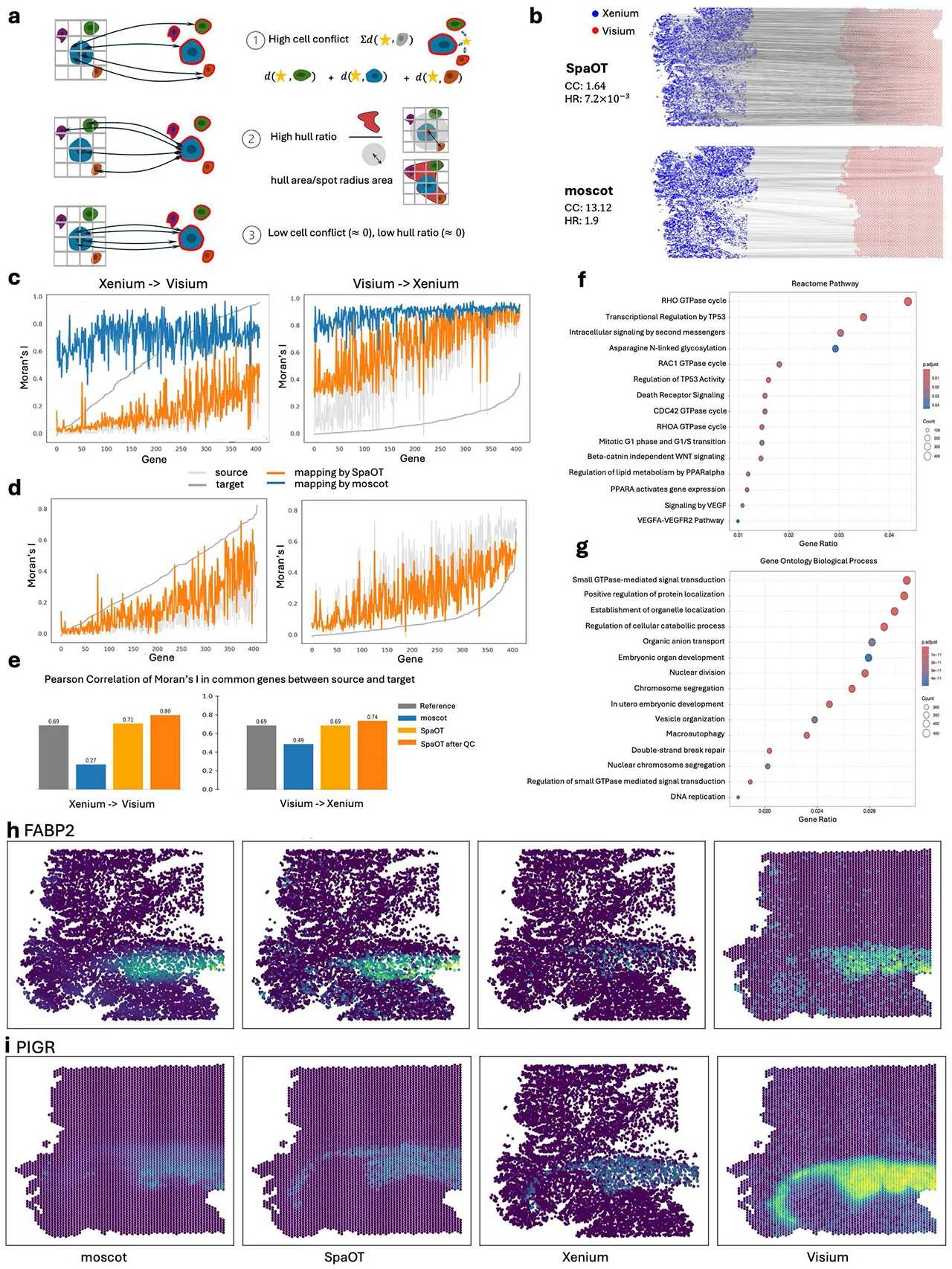
SpaOT performance on integrating spatial transcriptomics across different technology platforms. **a,** Schematic illustration of quantitative criteria for benchmarking alignment quality. Cell conflict quantifies how inconsistently each Visium spot is mapped across Xenium cells, whereas hull ratio measures how dispersed each Xenium cell is mapped to Visium spots. Accurate alignments are characterized by low cell conflict and low hull ratio values. **b,** Cross-platform alignment between Xenium and Visium spatial transcriptomics data from adjacent CRC tissue sections. Grey lines denote transport correspondences generated by SpaOT and moscot between Xenium cells and Visium spots. **c,** Line plots of spatial autocorrelation (Moran's I values) across 408 shared genes in bidirectional reconstruction tasks. **Left**: Xenium-to-Visium mapping. Dark grey, light grey, orange, and blue represent the reference Visium data, reference Xenium data, SpaOT-mapped Visium (transported from Xenium), and moscot-mapped Visium (transported from Xenium), respectively. **Right**: Visium-to-Xenium mapping. Dark grey, light grey, orange, and blue represent the reference Xenium data, reference Visium data, SpaOT-mapped Xenium (transported from Visium), and moscot-mapped Xenium (transported from Visium), respectively. **d,** Line plots of spatial autocorrelation (Moran’s I values) across 408 shared genes in bidirectional reconstruction tasks after quality-control filtering. **e,** Pearson correlation of Moran's I values for genes shared between the reference and mapped datasets. **Left**: Bars show the correlation between the reference Visium and (i) reference Xenium, (ii) moscot-mapped Visium (transported from Xenium), (iii) SpaOT-mapped Visium (transported from Xenium), and (iv) QC-filtered SpaOT-mapped Visium. **Right**: Bars show the correlation between the reference Xenium and (i) reference Visium, (ii) moscot-mapped Xenium (transported from Visium), (iii) SpaOT-mapped Xenium (transported from Visium), and (iv) QC-filtered SpaOT-mapped Xenium. **f,** Pathway enrichment analysis with Reactome pathways and **g,** Gene Ontology enrichment analysis of *SPP1*-associated genes on a SpaOT-mapped Xenium sample. **h,** Spatial expression patterns of the CRC-associated marker gene *FABP2* in the Xenium-to-Visium mapping task. Expression distributions reconstructed by moscot and SpaOT are shown alongside the original Xenium and Visium measurements. **i,** Spatial expression patterns of the immune-associated marker gene *PIGR* in the Visium-to-Xenium mapping task. Expression distributions reconstructed by moscot and SpaOT are shown alongside the original Visium and Xenium measurements. CRC: colorectal cancer, CC: cell conflict, HR: hull ratio.

Different spatial transcriptomics technologies offer complementary advantages in resolution and throughput, making cross-platform integration essential for comprehensive tissue characterization. We evaluated SpaOT on a colorectal cancer (CRC) study^36^ by aligning two adjacent tissue sections from the same donor (Sample P2 CRC) profiled using Visium and Xenium, respectively. Bidirectional mappings (Visium-to-Xenium and Xenium-to-Visium) were performed using identical parameter settings. Hull ratio, cell conflict, and Moran’s I^37^ were used to assess the alignment quality (Fig. 4a).

The resulting SpaOT mappings were spatially coherent, approximately parallel, and uniformly distributed between the Xenium and Visium sections (Fig. 4b). To assess biological fidelity in spatial autocorrelation, we compared Moran’s I of reconstructed genes with those observed in the target datasets (Fig. 4c). In the Xenium-to-Visium mapping task, SpaOT achieved 0.71, substantially outperforming moscot (0.27). Notably, this correlation slightly exceeded the intrinsic agreement between the original Xenium and Visium datasets ( 0.69 ), despite technical differences between the two platforms. Meanwhile, reconstructed genes remained highly consistent with the source Xenium sample with a Pearson correlation of 0.94, indicating that SpaOT preserves source spatial structure while accurately recovering target spatial organization. A similar pattern was observed for the Visium-to-Xenium reconstruction task, where SpaOT achieved a Pearson correlation of 0.69 with the target Xenium sample on Moran’s I, compared with 0.49 for moscot, while maintaining near-perfect agreement with the source Visium sample (correlation = 0.99).

We noticed that a subset of cells exhibited unusually low transcript counts or excessively high gene/UMI counts in the original dataset (**Supplementary Fig. 4**). Filtering with extra quality control further improved SpaOT performance, increasing Moran’s I correlation for bidirectional mappings (Fig. 4d, 4e).

To leverage the single-cell resolution of Xenium and transcriptome-wide coverage of Visium, Visium-to-Xenium mapping characterized the spatial expression patterns of *SPP1* gene identified in the original study^36^. Differential expression analysis^38^ between *SPP1*-enriched (top 20%) and *SPP1*-depleted (bottom 20%) regions revealed enriched pathways^39^ related to VEGF, TP53, WNT, and PPARA-associated lipid metabolism, indicating enhanced angiogenesis, proliferation, migration, and metabolic rewiring (Fig. 4f). Consistently, Gene Ontology^40^ (GO) analysis highlighted biological processes such as DNA replication, chromosome segregation, and nuclear division, suggesting increased proliferative and tumor-promoting activities in *SPP1*-enriched regions (Fig. 4g).

We further assessed whether the mapping preserved spatial patterns using CRC-associated marker genes (Fig. 4h). For *FABP2*, a gene associated with CRC progression^41^, SpaOT accurately preserved the spatial expression landscape while adapting it to the lower-resolution Visium domain. In contrast, moscot oversmoothed local spatial features and failed to recover the expression tail in the lower-left tissue region. Similarly, for *PIGR*, a gene linked to mucosal immunity and tumor microenvironment remodeling^42^, SpaOT faithfully transferred the expression pattern from Visium to the higher-resolution Xenium tissue architecture, whereas moscot lost several fine-scale spatial features (Fig. 4i).

### SpaOT robustly integrates spatial omics in multiple modalities

Next, we evaluated SpaOT on a spatial multi-omics mouse cerebellum dataset^43^ comprising spatial transcriptomics, spatial metabolomics, spatial proteomics reconstructed from adjacent tissue sections, and histological images corresponding to the transcriptomic section. Because these modalities measure distinct molecular entities with no overlapping features^44^, SpaOT employs a two-stage mapping strategy based on Canonical Correlation Analysis (CCA)^45^ to build anchors bridging the modalities (**Supplementary Fig. 5a**).

**Figure 5 ∣:**
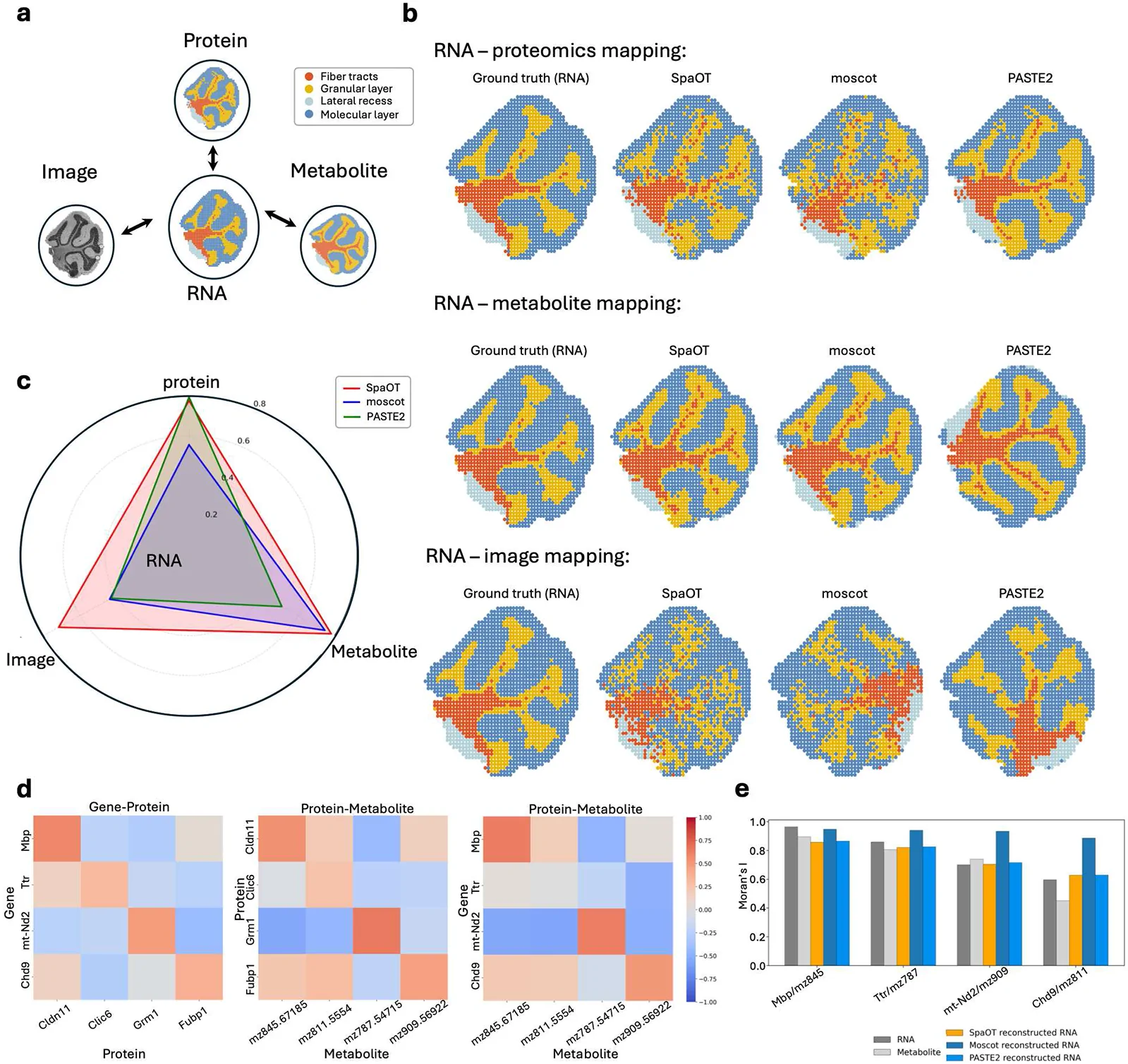
Performance of SpaOT integrating spatial transcriptomics with spatial proteomics, metabolomics, and histological imaging. **a,** Schematic overview of cross-modal integration in the mouse cerebellum dataset. Spatial transcriptomics (MAGIC-seq) was used as the anchor modality for alignment with spatial proteomics (PLATO), spatial metabolomics (MALDI-MSI), and histology imaging. **b,** Reconstruction results for cross-modal mapping tasks, including RNA-proteomics (top), RNA-metabolite (middle), and RNA-image (bottom) integration. Ground-truth RNA-defined tissue clusters (column 1) are compared with reconstructed cluster assignments generated by SpaOT (column 2), moscot (column 3), and PASTE2 (column 4). **c,** Spider plot of cross-modal cell type mapping accuracy across proteomics-to-transcriptomics, metabolomics-to-transcriptomics, and image-to-transcriptomics mapping tasks by SpaOT, moscot, and PASTE2. **d,** Cross-modal biological consistency analysis based on known co-localized molecular features. Heatmaps show Spearman correlations between genes, SpaOT-mapped proteins, and SpaOT-mapped metabolites across cerebellar tissue regions. **e,** Evaluation of spatial autocorrelation preservation using Moran’s I in metabolomics-to-transcriptomics reconstruction. Bar plots show Moran’s I for four colocalized gene-metabolite pairs in the original transcriptomics and metabolomics datasets, as well as for metabolites reconstructed and mapped onto the transcriptomics spatial coordinates by SpaOT, moscot, and PASTE2.

All the modalities were mapped to spatial transcriptomics, providing the highest quality, resolution, and molecular coverage (Fig. 5a). Across all mapping tasks, SpaOT consistently outperformed moscot and PASTE2 in preserving spatial patterns and biological identities (Fig. 5b, 5c).

To evaluate biological consistency, we examined molecular features reported to be spatially co-localized in the original study^43^. The gene *Mbp*, protein marker CLD11, and metabolite 845.68 m/z were all enriched within fiber tracts, while three additional groups of co-localized genes, proteins, and metabolites were enriched within the granular layer, lateral recess, and molecular layer. Integrating information across modalities produced clearer and more spatially distinct enrichment patterns than any individual modality alone (**Supplementary Fig. 5b**).

Cross-modal molecular consistency was further quantified by computing Spearman correlations between transcriptomic profiles and mapped proteins and metabolites at each spatial location (Fig. 5d). The observed correspondence between molecular signals and anatomical regions was highly consistent with known biological relationships reported in the original study.

Specifically, SpaOT and PASTE2 largely preserved spatial characteristics across modalities in the metabolomics-to-transcriptomics mapping task (Fig. 5e), while moscot suggested reduced sensitivity to modality-specific spatial variation by consistently producing elevated Moran’s I values. Together, these results demonstrate that SpaOT faithfully captures biologically meaningful cross-modal spatial relationships and enables robust integration of heterogeneous spatial omics modalities, even in the absence of direct feature overlap.

### SpaOT enables comprehensive spatial characterization of kidney research through Xenium and Visium HD integration

**Figure 6 ∣:**
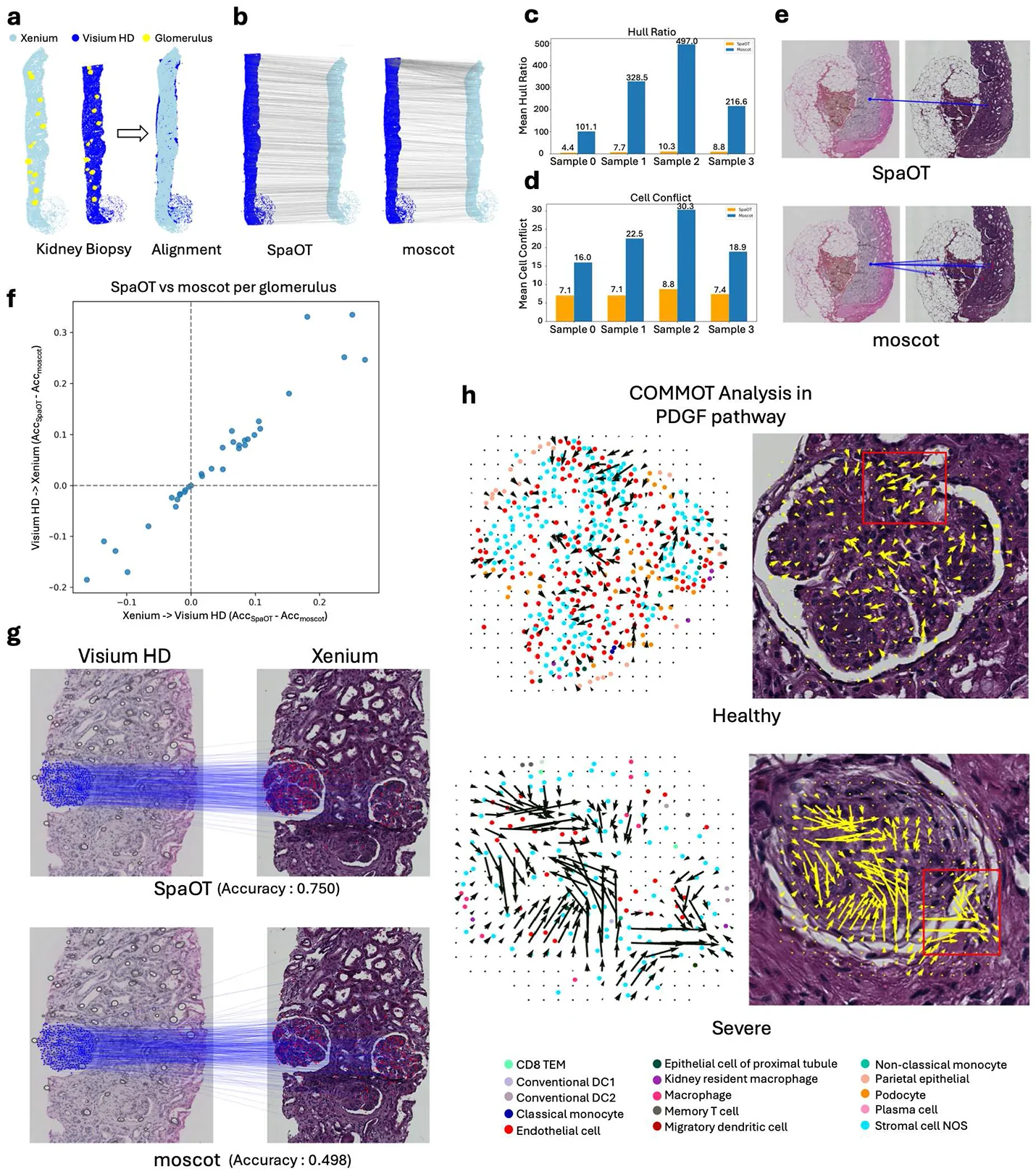
Integration of Xenium and Visium HD spatial transcriptomics in chronic kidney disease by SpaOT. **a,** A representative example (sample 2) of adjacent kidney biopsy sections profiled by Xenium (light blue) and Visium HD (dark blue). Glomerular regions are highlighted in yellow. On the right is the aligned result by SpaOT using Affine Transformation. **b,** Comparison of cross-platform mappings generated by SpaOT and moscot between Visium HD and Xenium sections. Straight lines in grey denote transport correspondences. Quantitative comparison of hull ratio (**c**) and cell conflict (**d**) for SpaOT and Moscot across four kidney biopsy samples. **e,** Representative comparison example of Visium HD-Xenium mapping from SpaOT and moscot visualized on aligned H&E images. **f,** Scatter plot of the relative performance differences of glomerulus mapping accuracy between SpaOT and moscot. The x-axis represents the performance improvement of SpaOT over moscot in Xenium-to-Visium HD mapping, whereas the y-axis represents the improvement in the reverse Visium HD-to-Xenium direction. Samples located in the first quadrant (up-right) indicate cases where SpaOT outperforms moscot mapping in both directions. **g,** One representative glomerulus mapping from Visium HD to Xenium with the overlaid histological images (Top: SpaOT, Bottom: moscot). **h,** Spatial signaling analysis of PDGF pathway activity within healthy (Top) and diseased (Bottom) glomerular regions on SpaOT-integrated data using COMMOT. Left, inferred signaling vector fields overlaid on glomerular cellular organization with annotated cell types. Right, corresponding histological regions from healthy and severely sclerotic glomeruli.

Chronic kidney disease (CKD) is a leading public health challenge affecting more than 850 million people worldwide^46^. We integrated Xenium and Visium HD spatial transcriptomics data from adjacent sections of four kidney biopsy samples from a CKD patient. We focused on glomeruli, the specialized capillary structures responsible for blood filtration and key pathological sites in CKD progression (Fig. 6a). With expert nephropathology annotation, 40 glomeruli were identified with sclerosis grades in severity.

In this cross-platform alignment task, SpaOT produced coherent, approximately parallel, and spatially localized correspondences between the Visium HD and Xenium sections (Fig. 6b). Quantitative evaluation using cell conflict and hull ratio confirmed better alignment quality of SpaOT across all biopsy samples (Fig. 6c, d). Similar performance was observed when restricted to glomerular regions (**Supplementary Fig. 6a**). The differences were further evident in aligned H&E images (Fig. 6e), where SpaOT predominantly mapped each Visium HD spot to a localized group of single cells in Xenium. Besides the whole-sample mapping, SpaOT accurately preserved glomerular structure while minimizing assignments outside the target glomerular region. SpaOT outperformed moscot in both mapping directions across most glomeruli (Fig. 6f, 6g).

Integrating Xenium with Visium HD data in glomerular regions, SpaOT enables whole-transcriptome analysis at *in-situ* single-cell resolution through Visium HD-to-Xenium mapping. In spatial signaling analysis with COMMOT^47^, the original Xenium or Visium HD data alone identified less abundant active signaling pathways. In contrast, SpaOT-integrated Xenium data revealed a substantially richer set of significant pathways associated with glomerular function and disease (**Supplementary Fig. 6b, c**).

We specifically examined PDGF signaling in spatial space (Fig. 6h). Compared to a healthy reference glomerulus, strong PDGF-mediated communication between endothelial and mesangial (stromal) cells extended from the glomerular hilum toward the glomerular tuft in a severe sclerosis glomerulus. These observations are consistent with the known role of PDGF signaling in mesangial cell proliferation, extracellular matrix remodeling, and progression of glomerulosclerosis during CKD^48^.

Likewise, CellChat^49^ detected far more ligand-receptor interactions following SpaOT integration than in the original Xenium data alone (**Supplementary Fig. 6d**). These interactions included key PDGF and VEGF signaling pathways implicated in mesangial activation, endothelial dysfunction, fibrosis, and tissue remodeling during CKD progression.

Together, cell-cell communication analyses revealed distinct communication landscapes between healthy and sclerotic glomeruli (**Supplementary Fig. 6e**). Diseased glomeruli displayed enhanced endothelial-mesangial (stromal) signaling associated with mesangial activation, extracellular matrix remodeling, and glomerulosclerosis, whereas healthy glomeruli maintained stronger and more diverse epithelial-immune and epithelial-stromal interactions. These results demonstrate that SpaOT preserves biologically meaningful cellular interactions and facilitates spatial communication analyses that provide new insights into complex kidney pathology.

## DISCUSSION

Integrating spatial omics across samples, platforms, and modalities remains challenging because of heterogeneity in molecular measurements, spatial resolution, cell composition, and noise characteristics. Defining a distance metric to faithfully measure sample-level discrepancy is the cornerstone of data integration. A metric on a space requires (i) non-negativity, (ii) identity of indiscernibles, (iii) symmetry, and (iv) triangle inequality^50^, providing a mathematically consistent notion of similarity. However, many existing unbalanced OT methods such as FUGW^51^ rely on KL divergence penalties and lack theoretical grounds in satisfying metric properties, potentially leading to distorted distance relationships. Consequently, downstream analyses such as clustering, prototype estimation, hierarchical comparison, and disease trajectory analysis lack rigorous geometric foundations. A central contribution of SpaOT is introducing the first semi-metric formulation for FPGW, a true metric under the total variation formulation (for q=1, Theorem 1 in Methods), enabling transport cost to function as a biologically meaningful sample distance for spatial omics. Although not a strict metric, SpaOT's semi-metric theoretically satisfies the first three criteria of a metric, but with a relaxed triangle inequality (**Supplementary Fig. 1b**). Our results demonstrate that the SpaOT-derived distance landscape faithfully reflects underlying biological dissimilarity, enabling robust biological comparisons ranging from disease stratification to multi-omics integration.

The semi-metric property of SpaOT represents both a theoretical and practical advance. Across colorectal and breast cancer cohorts, SpaOT-derived distances combined with lightweight ML classifiers consistently outperformed computationally heavy GNN approaches, which endure the information loss from pooling operations for compressing cell-level low-dimensional embeddings^52^. As a result, SpaOT’s formulation preserves fine-grained cellular neighborhood organization and cell-cell proximity patterns that are often diluted during representation learning^53,54^. These semi-metric advantages were particularly pronounced in distinguishing CRC subtypes and exhibited strong cross-cohort generalization in translational breast cancer studies, where SpaOT-derived distances are less sensitive to cohort-specific technical variation and batch effects.

Another distinctive feature of semi-metric is its ability to compute stable barycenters as mathematically meaningful representative tissues. We found that aggregating TMA cores into region-level and patient-level barycenters improved disease stratification performance, indicating they effectively reduce local noise while preserving biologically meaningful spatial and molecular signals. Specifically, barycenters provide interpretable prototypes that capture characteristic tissue organization, offering a principled framework for comparing phenotypes across multiple biological scales.

Beyond pairwise sample alignment, SpaOT demonstrated robust performance in reconstructing 3D tissue architectures from consecutive spatial transcriptomics sections. Because OT-based formulations are inherently invariant to global translation and rotation transformations (proof in **Supplementary Note D**), SpaOT avoids many of the alignment artifacts observed in representation-based approaches.

Given its formulation, SpaOT enables flexible and robust integration across spatial platforms and modalities, and supports downstream analyses such as differential expression, pathway enrichment, and cell-cell communication inference. In addition, SpaOT automatically determines the optimal amount of the transport mass, minimizing the need for prior knowledge of the mapping proportion, which is required by PASTE2^17^.

SpaOT still has several limitations. First, cross-modal integration currently relies on shared representations derived using auxiliary approaches such as CCA. The quality of these representations depends on the extent of biological correspondence between modalities and may be suboptimal when cross-modality correlations are weak. Second, SpaOT currently serves as a descriptive framework for measuring similarity and performing integration. Extending SpaOT into a generative framework^55^ could enable synthesis of intermediate tissue states while preserving spatial interpretability. Finally, the computation efficiency of SpaOT in large-scale studies will require further algorithmic optimization (**Supplementary Fig. 7**), including GPU acceleration, approximation schemes, and distributed computing.

More broadly, SpaOT suggests that mathematically consistent distance geometry, rather than alignment alone, may provide a general foundation for spatial multi-omics analysis. We anticipate that semi-metric OT will support not only integration but also atlas construction, disease progression modeling, foundation-model training, and spatial representation learning.

## METHODS

### High-level overview of SpaOT

We formulate spatial omics integration as an unbalanced optimal transport (OT) problem that maps mass from a source sample to a target sample, where source and target are arbitrary spatial omics samples. We first model a spatial omics sample as a metric-measure space (mm-space) in the context of measure theory, then introduce the Fused Partial Gromov-Wasserstein problem (FPGW) to preserve semi-metric characteristics in the OT framework. Next, we specialize the theoretical FPGW to a computationally practical discrete setting and propose two solvers: a Frank-Wolfe algorithm and a Sinkhorn algorithm.

### Modeling a spatial-omics sample as an mm-space

A spatial-omics sample (e.g., spatial transcriptomics, spatial proteomics, spatial metabolomics) with n units (cells, spots, or pixels) is modeled as an mm-space $X=(X,d_{X},\mu )$, where X is a compact set, e.g., $X={\left\{x_{i}\right\}}_{i=1}^{n}$ are the spatial units, each carrying a feature vector $\phi_{i}\in R^{p}$ (p features per unit) from molecular profiles. Depending on the modality, the features are gene expression within spatial transcriptomics, protein abundance in spatial proteomics, metabolite intensity in spatial metabolomics, and RGB values in images. $d_{X}$ is the structural distance between units, by default the Euclidean distance between spatial coordinates in the spatial omics sample. $\mu \in {ℳ}_{+}(X)$, where ${ℳ}_{+}(X)$ denotes the set of all finite positive Radon measures on X. By default, $\mu =\sum_{i=1}^{n}p_{i}\delta_{x_{i}}$ where $\delta_{x}$ is the Dirac Measure centered at x, $0<p_{i}<\infty$, ∀i. When $\sum_{i}p_{i}=1,\mu$ is a probability measure. The goal of SpaOT is to partially map the mass from the source sample X to the target sample Y, where the target sample $Y=(Y,d_{Y},\nu )$ is defined analogously.

### From optimal transport to fused partial Gromov-Wasserstein

SpaOT’s objective builds on a chain of classical transport problems, including optimal transport (OT) and its partial relaxation, Gromov-Wasserstein (GW), and their fused combination (FGW), which we introduce in turn before reaching the unbalanced, partial setting that defines the proposed FPGW. The details of the Optimal Transport problem’s background are in **Supplementary Notes A, B.**

#### Classical optimal transport.

Given a feature cost $C:X\times Y\to R_{+}$ that scores how expensive it is to match a source unit x to a target unit y (by default $C(x,y)={\left\|\;x-y\;\right\|}^{2}$), OT finds the coupling of minimal total cost ^14,15^:

(1) $$OT(\mu ,\nu )=\underset{\gamma \in \Gamma (\mu ,\nu )}{\text{min}}\int_{X\times Y}C\;(x,y)\;d\gamma (x,y)$$

where μ∈𝒫(X) and ν∈𝒫(Y) are probability measures and 𝒫(⋅) denotes the set of probability measures. The search space is the set of all couplings between μ and ν,

(2) $$\Gamma (\mu ,\nu )=\{\gamma \in 𝒫(X\times Y):\;\gamma_{1}=\mu ,\;\gamma_{2}=\nu \}$$

Here, $\gamma_{1}$, $\gamma_{2}$ are the first and second marginals of the joint measure γ. OT thus moves all of the source mass onto all of the target mass with the coupling. Importantly, classical OT requires equal total mass ∣μ∣=∣ν∣. When X=Y and $C(x,y)={\left\|\;x-y\;\right\|}^{p}$, $OT^{1∕p}$ is the *p*-Wasserstein distance, a metric on 𝒫(X).

#### Partial optimal transport (POT)^56^.

More practically, when the total mass of the two measures is not equal, there is no coupling exists in Γ(μ,ν). We define ∣σ∣=σ(X)=∫dσ as the total mass of a positive measure σ. POT relaxes the equal-mass constraint of classical OT by transporting only the mass as is worthwhile and leaving the rest mass unmatched, searching over the sub-couplings:

(3) $$\Gamma_{\leq }(\mu ,\nu )≔\{\gamma \in {ℳ}_{+}(X\times Y):\;\gamma_{1}\leq \mu ,\;\gamma_{2}\leq \nu \}$$

where $\gamma_{1}\leq \mu$ means $\gamma_{1}(B)\leq \mu (B)$ for every Borel set B, so part of each marginal may stay unmatched. POT then prices the unmatched mass linearly^56-58^:

(4) $$POT_{\lambda }(\mu ,\nu )=\underset{\gamma \in \Gamma_{\leq }(\mu ,\nu )}{\text{inf}}\int_{X\times Y}C\;(x,y)\;d\gamma (x,y)+\lambda (|\mu -\gamma_{1}|+|\nu -\gamma_{2}|)$$

The weight λ>0 controls the penalty for leaving mass unmatched. A match is rejected whenever its transport cost exceeds 2λ, making it more economical to leave the corresponding mass unmatched. The sub-couplings $\Gamma_{\leq }(\mu ,\nu )$ and this linear penalty are exactly the ingredients that FPGW carries over to the fused problem below.

#### Gromov-Wasserstein (GW).

When two samples reside in different spaces with no meaningful cross-domain feature cost, e.g., samples in different omics modalities, classical OT is not directly applicable. GW instead compares two samples through their internal geometry, matching correspondences that minimize discrepancies between pairwise distances measured within each sample^59^. We define $x^{\prime }$ as another unit adjacent to x within the same sample X, and $y^{\prime }$ as another unit adjacent to y within the same sample Y. With a structural loss L:R×R→R (by default $L(r_{1},r_{2})={|r_{1}-r_{2}|}^{2}$) and an exponent r≥1,

(5) $$GW(X,Y)=\underset{\gamma \in \Gamma (\mu ,\nu )}{\text{min}}\iint L\left(d_{X}^{r}(x,x^{\prime }),\;d_{Y}^{r}(y,y^{\prime })\right)d\gamma (x,y)\;d\gamma (x^{\prime },y^{\prime })$$

The quadratic (pairwise) term penalizes correspondences $\left(x,x^{\prime }\right)$ and $\left(y,y^{\prime }\right)$ whenever the source distance $d_{X}(x,x^{\prime })$ differs from the target distance $d_{Y}(y,y^{\prime })$. As a result, GW preserves the relational structure of the data rather than their absolute positions. With $L(r_{1},r_{2})={|r_{1}-r_{2}|}^{q}$, $GW^{1∕q}$ is a metric on mm-spaces, up to measure-preserving isometry.

#### Fused Gromov-Wasserstein (FGW).

Spatial omics units carry both a feature (molecular profile) and a position (spatial structure). FGW^60^ fuses them by adding the OT feature term (Eq. (1)) and the GW structure term (Eq. (5)) under a single transport plan, with weights $\omega_{1}$, $\omega_{2}$ to balance the intensities of both terms. $\omega_{1},\omega_{2}\geq 0$, $\omega_{1}+\omega_{2}=1$:

(6) $$FGW(X,Y)=\underset{\gamma \in \Gamma (\mu ,\nu )}{\text{min}}\omega_{1}\underset{OT\;term}{\underset{︸}{\int C(x,y)\;d\gamma (x,y)}}+\omega_{2}\underset{GW\;term}{\underset{︸}{\iint L\left(d_{X}^{r}(x,x^{\prime }),d_{Y}^{r}(y,y^{\prime })\right)d\gamma (x,y)\;d\gamma (x^{\prime },y^{\prime })}}$$

with C, $d_{X}$, $d_{Y}$ metrics and $L(r_{1},r_{2})={|r_{1}-r_{2}|}^{q}$, FGW is a semi-metric on mm-spaces, and a metric when q=1 ^60^.

#### Fused unbalanced GW (FUGW).

GW and FGW share OT’s equal-mass requirement, since they both search over the full couplings Γ(μ,ν) and transport every unit. The fused unbalanced GW (FUGW) of Thual et al.^51^ lifts this restriction by replacing the hard marginal constraints with f-divergence penalties^51^:

(7) $$FUGW_{\lambda }(X,Y)=\underset{\gamma \in {ℳ}_{+}(X\times Y)}{\text{inf}}\omega_{1}\int C(x,y)\;d\gamma +\omega_{2}\iint L(d_{X}^{r},d_{Y}^{r})\;d\gamma (x,y)d\gamma (x^{\prime },y^{\prime })\;\;+\lambda \left(D_{\phi_{1}}(\gamma_{1}^{⊗2}\;∥\;\mu^{⊗2})+D_{\phi_{2}}(\gamma_{2}^{⊗2}\;∥\;\nu^{⊗2})\right)$$

where $D_{\phi_{1}}$, $D_{\phi_{2}}$ are f-divergences and the search now ranges over all positive measures ${ℳ}_{+}(X\times Y)$, and $\mu^{⊗2}$ is the product measure defined by $d\mu^{⊗2}(x,x^{\prime })=d\mu (x)\;d\mu (x^{\prime })$.

#### Fused partial GW (FPGW).

Practical spatial-omics samples differ in cell/spot counts and resolution, and contain diverse types of outliers, so forcing a full match misroutes mass onto noise. Our FPGW is the special case of FUGW in which both f-divergences are taken to be the total-variation distance. This choice imposes a linear penalty on unmatched mass, such that a unit of mass is matched whenever the transport cost is below its threshold and left unmatched otherwise, thereby directly controlling how much of the sample is transported. Reusing the feature cost C and structural loss L, the FPGW is defined as

(8) $$FPGW_{\lambda }(X,Y)=\underset{\gamma \in {ℳ}_{+}(X\times Y)}{\text{inf}}\omega_{1}\int C(x,y)\;d\gamma (x,y)+\omega_{2}\iint L\left(d_{X}(x,x^{\prime }),d_{Y}(y,y^{\prime })\right)d\gamma (x,y)\;d\gamma (x^{\prime },y^{\prime })\;\;+\lambda \left({\left\|\;\mu^{⊗2}-\gamma_{1}^{⊗2}\;\right\|}_{TV}+{\left\|\;\nu^{⊗2}-\gamma_{2}^{⊗2}\;\right\|}_{TV}\right)$$

Here ${\left\|\cdot \right\|}_{TV}$ is the total-variation (TV) norm, where for a signed Radon measure ρ, ${\left\|\rho \right\|}_{TV}=|\rho_{+}|+|\rho_{-}|$ and $\left(\rho_{+},\rho_{-}\right)$ are positive Radon measures obtained by the Hahn-Jordan decomposition of ρ, i.e., $\rho =\rho_{+}-\rho_{-}$.

Exactly as POT reduces to sub-couplings, we found that computing FPGW in Eq. (8) can be simplified by restricting all of ${ℳ}_{+}(X\times Y)$ to the sub-couplings $\Gamma_{\leq }(\mu ,\nu )$, as Proposition 1.

##### Proposition 1 (Reduction of FPGW to sub-couplings).

If C(x,y)≥0 for all x, y and $L(d_{X}^{r},d_{Y}^{r})\geq 0$, then Eq. (8) is equivalent to

(9) $$FPGW_{\lambda }(X,Y)=\underset{\gamma \in \Gamma_{\leq }(\mu ,\nu )}{\text{min}}\omega_{1}\int C(x,y)\;d\gamma (x,y)\;\;+\omega_{2}\iint L\left(d_{X}^{r}(x,x^{\prime }),d_{Y}^{r}(y,y^{\prime })\right)d\gamma (x,y)\;d\gamma (x^{\prime },y^{\prime })\;\;+\lambda ({|\mu |}^{2}+{|\nu |}^{2}-2{|\gamma |}^{2}).$$

With Eq. (9), the search reduces to the sub-couplings $\Gamma_{\leq }(\mu ,\nu )$, and the two TV penalties collapse to the closed-form term $\lambda ({|\mu |}^{2}+{|\nu |}^{2}-2{|\gamma |}^{2})$. Full Proof and detailed discussion in **Supplementary Notes C**. We further prove FPGW theoretically admits a minimizer in Proposition 2.

##### Proposition 2 (Existence of a minimizer).

For mm-spaces X, Y with X, Y compact, the FPGW problem in Eq. (8) (equivalently Eq. (9)) admits a minimizer; that is, the infimum is attained.

Since there exists a minimizer, the solvers discussed at the end of this section are well-posed for computing. The proof is in **Supplementary Notes C**.

Finally, like OT, GW, and FGW, the FPGW objective behaves like a distance.

##### Theorem 1 (Semi-metric property of FPGW).

Take $C(x,y)={\left\|\;x-y\;\right\|}^{q}$, $L(r_{1},r_{2})={|r_{1}-r_{2}|}^{q}$, and $\omega_{2},\lambda >0$. Then the (mass-normalized) FPGW is a semi-metric on mm-spaces, up to measure-preserving isometry; when q=1 it is a metric.

FPGW is symmetric, non-negative, and vanishes exactly between equivalent spaces, and for q=1 it satisfies the triangle inequality, the properties that FUGW with a KL penalty is not known to have. The full proofs are in **Supplementary Notes C-D**.

Furthermore, an equivalent mass-constrained formulation (FMPGW), in which the penalty λ plays the role of the Lagrange multiplier of the transported-mass constraint, is also developed, and we further discuss how FPGW relates to existing transport problems (Gromov-Wasserstein, classical OT, and fused GW) in **Supplementary Notes K**.

### Frank-Wolfe Solver

For solving the problem of FPGW, we work in the discrete setting throughout with $\mu =\sum_{i=1}^{n}p_{i}\delta_{x_{i}}$ and $\nu =\sum_{j=1}^{m}q_{j}\delta_{y_{j}}$, $p\in R_{+}^{n}$, $q\in R_{+}^{m}$. We define a transport plan as a non-negative matrix $\gamma \in R_{+}^{n\times m}$, and the sub-coupling condition becomes a row/column-sum constraint,

(10) $$\Gamma_{\leq }(p,q)=\{\gamma \in R_{+}^{n\times m}:\;\gamma 1_{m}\leq p,\;\gamma^{T}1_{n}\leq q\}$$

To facilitate computational purposes in a discrete setting, the two cost terms in Eq. (8) can be written as matrix/tensor expressions: The feature-cost matrix $C=[C(x_{i},y_{i})]\in R^{n\times m}$ turns the OT term ∫C(x,y)dγ(x,y) into the Frobenius inner product $\langle C,\gamma \rangle ≔\sum_{i,j}C_{ij}\gamma_{ij}$. Similarly, from the intra-sample distance matrices $C^{X}=[d_{X}^{r}(x_{i},x_{i^{\prime }})]\in R^{n\times n}$ and $C^{Y}=[d_{Y}^{r}(y_{j},y_{j^{\prime }})]\in R^{m\times m}$, we form the structure tensor $M_{i,j,i^{\prime },j^{\prime }}={\left[d_{X}^{r}(x_{i},x_{i^{\prime }})-d_{Y}^{r}(y_{j},y_{j^{\prime }})\right]}^{2}$, $M\in R^{n\times m\times n\times m}$, which stores all pairwise loss values within the source (i and $i^{\prime }$) and target (j and $j^{\prime }$), so the GW term $\gamma^{⊗2}(L)$ becomes the quadratic form $\sum_{i,j,i^{\prime },j^{\prime }}M_{i,j,i^{\prime },j^{\prime }}\gamma_{i,j}\gamma_{i^{\prime },j^{\prime }}$. Then the FPGW problem in Eq. (9) converts to a more computationally friendly version Eq. (11)

(11) $$FPGW_{\lambda }(X,Y)=\underset{\gamma \in \Gamma_{\leq }(p,q)}{\text{min}}\underset{{ℒ}_{C,M-2\lambda ∕\omega_{2}}}{\underset{︸}{\omega_{1}\langle C,\gamma \rangle +\omega_{2}\langle (M-2\lambda ∕\omega_{2})\;\circ \;\gamma ,\gamma \rangle }}+\underset{\text{constant}}{\underset{︸}{\lambda ({|\mu |}^{2}+{|\nu |}^{2})}}$$

The additive constant $\lambda \omega_{2}({|p|}^{2}+{|q|}^{2})$ can be dropped for it does not influence the results of the transport plan. So solving Eq. (9) leads to a quadratic program, where M−2λ subtracts 2λ from each entry of M (folding the $-2\lambda {|\gamma |}^{2}$ penalty into the structure term), and ${\left[M\;\circ \;\gamma \right]}_{ij}=\sum_{i^{\prime },j^{\prime }}M_{i,j,i^{\prime },j^{\prime }}\gamma_{i^{\prime },j^{\prime }}$ is the tensor product contraction of M against γ, which is the quantity that drives the gradient calculation below. The objective is non-convex but quadratic, and we use the Frank-Wolfe scheme^61^ as a conditional-gradient solver.

#### Gradient and direction step.

At iteration k−1, the gradient of $ℒ≔{ℒ}_{C,M-2\lambda }$ at the current plan $\gamma^{\left(k-1\right)}$ is

(12) $$\nabla (\gamma^{\left(k-1\right)})=\omega_{1}\;C+\omega_{2}\;(M+M^{T}-4\lambda ∕\omega_{2})\;\circ \;\gamma^{\left(k-1\right)}$$

where the cross-term doubles because γ appears on both sides of the quadratic form 〈M∘γ,γ〉, and the tensor transpose $M_{i,j,i^{\prime },j^{\prime }}^{T}≔M_{i^{\prime },j^{\prime },i,j}$ captures the other-side contraction. Instead of solving the original objective Eq. (11), Frank-Wolfe algorithm solves the first order Taylor expansion:

(13) $$\gamma^{(k)\prime }=\text{arg}\underset{\gamma \in \Gamma_{\leq }(p,q)}{\text{min}}\langle \nabla ℒ(\gamma^{\left(k-1\right)}),\;\gamma \rangle$$

which can be converted to a POT problem and thus can be solved exactly by a linear program or approximately by entropic (Sinkhorn) iterations (**Supplementary Notes I**).

#### Line search algorithm.

We then choose the optimal step size $\alpha^{*}\in [0,1]$,

(14) $$\alpha^{*}≔\text{arg}\underset{\alpha \in [0,1]}{\text{min}}{ℒ}_{C,M-2\lambda ∕\omega_{2}}\left((1-\alpha )\gamma^{\left(k-1\right)}+\alpha \gamma^{(k)\prime }\right)$$

The term $\alpha^{*}$ is given by the following:

α∗={1ifa≤0,a+b≤0,0ifa≤0,a+b>0,clip(−b∕2a,[0,1])ifa>0,}

where $\delta \gamma =\gamma^{(k)\prime }-\gamma^{\left(k-1\right)}$, $a=\omega_{2}\langle (M-2\lambda ∕\omega_{2})\;\circ \;\delta \gamma ,\delta \gamma \rangle$, and $b=\langle \nabla ℒ(\gamma^{\left(k-1\right)}),\delta \gamma \rangle$ to reuse the gradient. Clip is the function that clip(x,[a,b])=min(max(x,a),b).

Formally, we have Frank-Wolfe algorithm for FPGW as follows:

**Table:** 

Algorithm 1: Frank-Wolfe Algorithm for FPGW
$$\begin{array}{cc} \; & \;\mathrm{Input}:C\in R^{n\times m},C^{X}\in R^{n\times n},C^{Y}\in R^{m\times m},p\in R_{+}^{n},q\in R_{+}^{m},\omega_{2}\in [0,1],\lambda ∕\omega_{2}\geq 0\\ \; & \;\mathrm{Output}:\gamma^{\left(final\right)}\\ \; & \;\mathrm{for}\;k=1,2,\ldots \mathrm{do}\\ \; & \;\;\;\text{Compute gradient:}\;G^{\left(k\right)}\leftarrow \omega_{1}C+\omega_{2}(M+M^{T}-4\lambda ∕\omega_{2})\;\circ \;\gamma^{\left(k\right)}\\ \; & \;\;\;\text{Solve partial-OT direction:}\;\gamma^{{\left(k\right)}^{\prime }}\leftarrow \text{arg}\underset{\gamma \in \Gamma_{\leq }(p,q)}{\text{min}}{\langle G^{\left(k\right)},\gamma \rangle }_{F}\\ \; & \;\;\;\text{Line search:}\;\text{Compute}\;\alpha^{\left(k\right)}\in [0,1]\;\text{from the closed-form}\;\alpha^{*}\;\text{above}.\\ \; & \;\;\;\text{Update plan:}\gamma^{\left(k+1\right)}\leftarrow (1-\alpha^{\left(k\right)})\gamma^{\left(k\right)}+\alpha^{\left(k\right)}+\alpha^{\left(k\right)}\gamma^{{\left(k\right)}^{\prime }}\\ \; & \;\;\;\text{if convergence},\;\text{break}\\ \; & \;\text{end for}\\ \; & \;\text{return}\;\gamma^{\left(final\right)}\leftarrow \gamma^{\left(k\right)} \end{array}$$

### Complexity analysis.

#### Computation of gradient.

A naive contraction M∘γ costs time complexity of $𝒪(n^{2}m^{2})$, when the structural loss L factorizes^62^ as

$$L(r_{1},r_{2})=f_{1}(r_{1})+f_{2}(r_{2})-h_{1}(r_{1})h_{2}(r_{2}),$$,

e.g. $L={|r_{1}-r_{2}|}^{2}$ with $f_{1}(r)=f_{2}(r)=r^{2}$, $h_{1}(r)=2r$, $h_{2}(r)=r$. This complexity can be reduced to $𝒪(n^{2}+m^{2})$ via

(15) $$M\;\circ \;\gamma =f_{1}(C^{X})\gamma_{1}1_{m}^{T}+1_{n}\gamma_{2}^{T}f_{2}(C^{Y})-h_{1}(C^{X})\gamma \;h_{2}(C^{Y})$$

This reduced complexity allows the solver to scale to large-scale samples at the atlas level, where the gradient term ∇ℒ in Eq. (12) can be solved in quadratic time.

#### Complexity of each FW iteration.

Because the line search step has a closed-form solution and the gradient term can be computed in quadratic time, in each Frank-Wolfe iteration, the computation is dominated by the Partial OT step in Eq. (13). In particular, the time complexity is 𝒪((n+m)nm) with a linear-programming Solver, or $𝒪\left(\frac{1}{\epsilon }\;\text{ln}(n+m)\;nm\right)$ with a Sinkhorn OT solver.

#### Number of iteration steps of FW algorithm.

To analyze the required iteration numbers of convergence, in particular, convergence is measured by the Frank-Wolfe gap^61^:

$$g_{k}=\max_{\gamma \in \Gamma_{\leq }(p,q)}\langle \nabla ℒ(\gamma^{\left(k\right)}),\gamma^{\left(k\right)}-\gamma \rangle \geq 0$$

which is zero exactly at a stationary plan as Theorem 2. The detailed derivations of the gradient, line search, and the rate below are in **Supplementary Sections F-H**.

##### Theorem 2 (Convergence of the Frank-Wolfe solver).

For the quadratic loss $L(r_{1},r_{2})={|r_{1}-r_{2}|}^{2}$, the minimal Frank-Wolfe gap of Algorithm 1 after K iterations satisfies

$$\underset{1\leq k\leq K}{\text{min}}g_{k}\leq \frac{\max\{2L_{1},\;4\omega_{2}\;\text{min}{\left(|p|,|q|\right)}^{2}nm\;\max(2{\left(C^{X}\right)}^{2}+{2(C^{Y})}^{2},\;2\lambda ∕\omega_{2})\}}{\sqrt{K}},$$

where $L_{1}$ is the initial sub-optimality. Hence the rate is $O(1∕\sqrt{K})$, i.e., at most $O(1∕\epsilon^{2})$ iterations reach an ϵ-stationary plan.

To clarify, all of the analysis in this paper uses the Frank-Wolfe solver.

### Sinkhorn Solver

Another popular solver for the Gromov-Wasserstein problem is the Sinkhorn algorithm^63^. This GPU-friendly solver adds entropic regularization with weight ϵ>0 and relative entropy H(A∥B)=∫log(dA∕dB)dA for any positive Radon measures A and B. Using the Sinkhorn solver, we define FPGW in Eq. (9) as

(16) $$FPGW^{entropic}(X,Y)≔\underset{\gamma \in \Gamma_{\leq }(\mu ,\nu )}{\text{min}}ℒ(\gamma )+\epsilon H\left(\gamma^{⊗2}\;∥\;{\left(\mu \;⊗\;\nu \right)}^{⊗2}\right)\;\;ℒ(\gamma )=\omega_{1}\langle C,\gamma \rangle +\omega_{2}\langle L(d_{X}^{r},d_{Y}^{r}),\gamma^{⊗2}\rangle +\lambda ({|\mu |}^{2}+{|\nu |}^{2}-2{|\gamma |}^{2}).$$

Because the structure term is not jointly convex, directly optimizing the objective is challenging. To obtain a tractable optimization problem, we introduce an auxiliary transport plan π and relax the problem into a bilinear form involving two plans γ, π. This yields a lower-bound formulation denoted $\text{LB-}{\text{FPGW}}_{\lambda }$ (lower bound of Fused Partial Gromov Wasserstein), which provides a lower bound on FPGW entropic:

(17) $$\;LB-FPGW_{\lambda }(X,Y)=\underset{\gamma ,\pi \in \Gamma_{\leq }(\mu ,\nu )}{\text{min}}ℱ(\gamma ,\pi )+\epsilon H\left(\pi \;⊗\;\gamma \;∥\;{\left(\mu \;⊗\;\nu \right)}^{⊗2}\right)\;ℱ(\gamma ,\pi )≔\omega_{1}\langle d(x,y),\frac{\gamma +\pi }{2}\rangle +\omega_{2}\langle L(d_{X}^{r},d_{Y}^{r}),\gamma \;⊗\;\pi \rangle +\lambda ({|\mu |}^{2}+{|\nu |}^{2}-2|\gamma ||\pi |).$$

The resulting objective is optimized efficiently by alternating updates of γ and π. For fixed π, each subproblem is an entropic partial OT problem (proof in **Supplementary Section I**) which can be defined as:

(18) $$\;\underset{\gamma \in \Gamma_{\leq }(\mu ,\nu )}{\text{min}}\int c_{\pi }\;d\gamma +\lambda |\pi |(|\mu |+|\nu |-2|\gamma |)+\epsilon |\pi |\;H(\gamma \;∥\;\mu \;⊗\;\nu )\;c_{\pi }(x,y)=1∕2\omega_{1}d(x,y)+\omega_{2}\;[L(d_{X}^{r},d_{Y}^{r})\;\circ \;\pi ](x,y)+\epsilon \;H(\pi \;∥\;\mu ⊗\;\nu ).$$

Given these fundamental results, we can present the Sinkhorn algorithm for FPGW as Algorithm 2.

**Table:** 

Algorithm 2: Sinkhorn Algorithm for FPGW
$$\begin{array}{cc} \; & \;\mathrm{Input}:C\in R^{n\times m},C^{X}\in R^{n\times n},C^{Y}\in R^{m\times m},p\in R_{+}^{n},q\in R_{+}^{m},\omega_{2}\in [0,1],\lambda \geq 0\\ \; & \;\mathrm{Output}:\gamma \\ \; & \;\mathrm{for}\;k=1,2,\ldots \mathrm{do}\\ \; & \;\;\;\pi \leftarrow \gamma \\ \; & \;\;\;\text{Solve the Sinkhorn partial OT problem:}\\ \; & \;\;\;\gamma \leftarrow \underset{\gamma \in \Gamma_{\leq }(\mu ,\nu )}{\text{min}}\int_{X\times Y}c_{\pi }(x,y)d\gamma +\lambda |\pi |(|\mu |+|\nu |-2|\gamma |)+\epsilon |\pi |H(\gamma \;∥\;\mu \;⊗\;\nu )\\ \; & \;\;\;\text{Rescale}\;\pi \leftarrow \sqrt{|\gamma |∕|\pi |}\pi \\ \; & \;\;\;\text{Fix}\;\gamma \;\text{and solve the similar Sinkhorn partial OT problem:}\\ \; & \;\;\;\pi \leftarrow \underset{\pi \in \Gamma_{\leq }(\mu ,\nu )}{\text{min}}\int_{X\times Y}c_{\gamma }(x,y)d\pi +\lambda |\gamma |(|\mu |+|\nu |-2|\pi |)+\epsilon |\gamma |H(\pi \;∥\;\mu \;⊗\;\nu )\\ \; & \;\;\;\text{Rescale}\;\gamma \leftarrow \sqrt{|\pi |∕|\gamma |}\gamma \\ \; & \;\;\;\\ \; & \;\;\;\text{Break if}\;\pi \approx \gamma \\ \; & \;\text{end}\;\text{for} \end{array}$$

### FPGW barycenter

For a total number of K omics samples, $X^{k}=(X^{k},p^{k},C^{k})$, k=1,…,K, represents K mm-spaces, where $X^{k}\in R^{n_{k}\times d}$ is the feature matrix, $p^{k}\in R_{+}^{n_{k}}$ is the mass distribution, and $C^{k}\in R^{n_{k}\times n_{k}}$ is the structural matrix. Given weights $\beta_{k}\geq 0$ satisfying $\sum_{k=1}^{K}\beta_{k}=1$, a target mass profile $p\in R_{+}^{n}$, and a penalty parameter $\lambda_{k}$, the FPGW barycenter is the representative mm-space X=(X,p,C), where the feature matrix $X\in R^{n\times d}$ and structure matrix $C\in R^{n\times m}$, that minimizes the weighted FPGW discrepancy as Eq. (19)

(19) $$\underset{C,X}{\text{min}}\sum_{k=1}^{K}\beta_{k}\;FPGW_{L,\lambda_{k}}(X,X^{k})=\underset{C,X}{\text{min}}\underset{\gamma^{k}\in \Gamma_{\leq }(p,p^{k})}{\text{min}}\sum_{k=1}^{K}\left(\omega_{1}^{k}\langle D(X,X^{k}),\gamma^{k}\rangle +\omega_{2}^{k}\langle L(C,C^{k}),{\left(\gamma^{k}\right)}^{⊗2}\rangle +\lambda_{k}\left({|p^{k}|}^{2}+{|p|}^{2}-2{|\gamma^{k}|}^{2}\right)\right)$$

where $D(X,X^{k})=\left[{\left\|x_{i}-x_{j}^{2}\right\|}^{2}\right]$. Fixing the transport plans (i.e., ${\left\{\gamma^{k}\right\}}_{k=1}^{K}$) makes the objective convex in the barycenter variables (C, X), whereas we can update the transport plans via FPGW solvers (Frank-Wolfe or Sinkhorn) by fixing (C, X) . Therefore, we optimize it using an alternating minimization procedure that iteratively updates (C, X) and the transport plans.

The feature update uses the barycentric projection of each sample onto the barycenter support,

x^ik={1γ1k[i]∑jγi,jkxjkifγ1k[i]>0,xiifγ1k[i]=0,}

and the optimal feature X is obtained by

$$X={\left[x_{1},\cdots ,x_{n}\right]}^{T},x_{i}=\frac{\sum_{k-1}^{K}\beta_{k}\gamma_{1}^{k}[i]\;{\hat{x}}_{i}^{k}}{\sum_{k=1}^{K}\beta_{k}\gamma_{1}^{k}[i]},\forall i\in [1:n],with\frac{0}{0}=0.$$

In addition, the structure update has the closed form (for $L(x,y)={|x-y|}^{2}$)

$$C=\frac{\sum_{k}\beta_{k}\;\gamma^{k}\;C^{k}{\left(\gamma^{k}\right)}^{T}}{\sum_{k}\beta_{k}\;\gamma_{1}^{k}{\left(\gamma_{1}^{k}\right)}^{T}}.$$

**Table:** 

Algorithm 3: FPGW Barycenter
$$\begin{array}{cc} \; & \;\mathrm{Input}:\text{mm-spaces}\;{\left\{(X^{k},p^{k},C^{k})\right\}}_{k=1}^{K},\;\text{weights}\;\beta_{k},\;\text{target mass}\;p,\;\text{penalties}\;\lambda_{k},\;(\omega_{1}^{k},\omega_{2}^{k}).\\ \; & \;\mathrm{Output}:\text{barycenter}\;X=(X,p,C).\\ \; & \;\text{Initialize feature matrix}\;X\;\text{and structure matrix}\;C.\\ \; & \;\mathrm{for}\;t=1,2,\ldots \mathrm{do}\\ \; & \;\;\;\text{For each}\;k:\text{update}\;\gamma^{k}\;\text{by solving}\;FPGW_{\lambda_{k}}(X,X^{k})\;\text{with Algorithm 1 or 2}.\\ \; & \;\;\;\text{Update structure}\;C\;\text{via the closed-from structure update above}.\\ \; & \;\;\;\text{Update features}\;X\;\text{via the barycentric-projection update above}.\\ \; & \;\;\;\text{if converged},\;\text{break}.\\ \; & \;\text{end for}\\ \; & \;\mathrm{return}\;X=(X,p,C). \end{array}$$

The derivation of updates in the general loss case and the optimality proof of the feature update was given in **Supplementary Section J**. SpaOT uses Algorithm 3 to compute cluster centroids in the OT-based k-means framework and the representative samples in the disease-stratification analyses.

### Noise simulation and benchmarking

To evaluate SpaOT’s robustness against noise, synthetic crescent-shaped point clouds were generated from a two-dimensional two-moons distribution using the implementation provided in *scikit-learn*^64^. To increase variability in both node features and structural complexity, additional vertically shifted replicas were generated for both the source and target moons. One-hot encodings of two distinct node labels were used as node features.

Both outlier noise and structural noise were introduced in benchmarking. For outlier noise, a group of extra points (10%) under a Gaussian distribution was generated outside of the target point cloud. So the maximum error rate is bounded by 9.09% (10%/110%). For structural noise, the target moon geometry was progressively interpolated from its original crescent shape toward a full moon while preserving the layered node-feature arrangement. All simulations were conducted using fixed random seeds to ensure reproducibility.

All benchmarked methods followed the same processing pipeline. For SpaOT, moscot, PASTE2, and PythonOT, pairwise Euclidean distances between spatial coordinates were used to characterize structural relationships. All remaining parameters were set to their default or recommended configurations, with detailed settings provided in the Parameter Settings section. For 3d-OT, we followed the official protocol in which the source and target moon datasets were first converted into graph representations using k-nearest-neighbor connectivity and one-hot node features. Two graph encoders were independently trained on both source and target to learn latent geometric representations. Then the structural and feature information across graphs was aligned on the learnt representations.

**Simulation for Graph Clustering** was followed by the synthetic dataset generation and benchmarking pipeline proposed by Vayer et al.^65^ Three graph categories containing one, two, or three communities were generated, and each category contains five graph instances according to a stochastic block model (SBM). Each graph instance consisted of 30-40 nodes, and node feature values as single-value vectors were randomly sampled.

To assess the model robustness, outliers as 30% additional nodes were added to half of the graph instances in each category, where features were sampled from a shifted distribution (minimum value = 50) to separate them from the original nodes. Each outlier node was randomly connected to an existing node in the graph. Fixed random seeds were used for reproducibility.

We benchmarked OT-derived transport cost as the distance for OT-based methods, including SpaOT, moscot, PASTE2, and PythonOT, in the graph clustering framework based on K-means algorithm. Given graphs $\left\{G_{1},\ldots ,G_{K}\right\}$ and the number of clusters $K^{\prime }$, here K=15, $K^{\prime }=3$, the clustering is iterated in two steps: (i) computing the discrepancy between each graph and the centroid and assigning each graph to its closest centroid, and (ii) updating each centroid as the corresponding barycenter of the graphs assigned to the cluster.

Graph barycenters were employed for computing the cluster centroid. SpaOT used the proposed FPGW barycenter, while PythonOT used the standard FGW barycenter. As moscot and PASTE2 lack native barycenter implementations, moscot was paired with PythonOT's FGW barycenter with the balanced setting and PASTE2 with SpaOT's FPGW barycenter with the partial setting.

To compute the discrepancies, each graph G=(V,E) is represented as an mm-space $X=\left(V,C,\sum_{i=1}^{N}\frac{1}{N_{G}}\delta_{V_{i}}\right)$, where $C=[c(v_{i},v_{i^{\prime }})]$ denotes the pairwise shortest-path distance matrix induced by E, and $N_{G}$ denotes the number of non-outlier nodes and is specified according to the experimental setup.

The centroid update is defined through the corresponding FGW/FPGW barycenter formulation.

$$\underset{G}{\text{min}}\sum_{G_{k}\in G^{k^{\prime }}}\frac{1}{|G^{k^{\prime }}|}D_{\lambda_{k}}(G,G_{k}),$$

where $D_{\lambda_{k}}$ denotes the FGW or FPGW discrepancy depending on the method, and $G^{k^{\prime }}$ denotes the set of graphs assigned to cluster $k^{\prime }$. The two steps are repeated until convergence of the cluster assignments or centroids.

### Disease stratification in supervised learning

#### Intra-dataset stratification.

For each spatial omics dataset, we constructed mm-spaces and computed pairwise sample distances using SpaOT. The resulting SpaOT distance matrices between each cell or spot in the source and each cell or spot in the target were used as inputs to machine learning classifiers under a 5-times repeated 3-fold cross-validation scheme. For GNN-based baselines, spatial graphs and GNN models were constructed following the established protocols^27^ using k-nearest-neighbor connectivity, with edge weights defined by spatial proximity and node features by molecular profiles. Graph-level predictions were obtained through the same protocol using GNN layers, pooling, and a classification head. The same cross-validation protocol was applied to all GNN models.

#### Inter-dataset stratification.

For spatial transcriptomics data across cohorts METABRIC and Jackson, Principal Component Analysis (PCA)^66^ was applied for dimensionality reduction on the raw distance matrix between each sample, and only 27 shared proteomic markers were retained and aligned across cohorts. For SpaOT, models were trained using intra-dataset distance matrices from the training dataset and tested using inter-dataset distance matrices between training and testing samples. For GNN baselines, spatial graphs were constructed as above, with models trained on one dataset and evaluated on an independent dataset.

### 3D Tissue Reconstruction

We first constructed metric measure spaces from the normalized molecular profiles and spatial structural information of each tissue section. SpaOT was then applied sequentially to adjacent sections to obtain transport plans that establish cross-section correspondences. Based on these transport plans, neighboring sections are aligned using affine (rigid) transformations, enabling reconstruction of the z-axis and generation of a three-dimensional tissue reconstruction.

Given source and target spatial omics samples with spatial coordinates $X_{s}$ and $X_{t}$, and an optimal transport plan γ obtained from SpaOT, we estimate an affine transformation to align the target sample to the source sample. The transport-weighted source coordinates were used to compute a cross-covariance matrix, from which the optimal rotation matrix (R) is estimated via singular value decomposition (SVD) following the orthogonal Procrustes formulation. A translation vector (t) is subsequently computed to align the centroids, yielding the transformed target coordinates $\hat{X_{t}}=RX_{t}+t$ for the full target sample.

### Mapping between Same Omics and Different Technology

For cross-technology mapping within the same omics modality (e.g., Xenium-Visium mapping), we identified shared anchors between datasets, such as shared molecular markers (e.g., genes) measured across platforms. In data integration, only the shared markers were retained and subsequently min-max normalized to be included in computing the OT term in Eq. (1). For the GW term in Eq. (5), pairwise spatial Euclidean distances were computed and min-max normalized between [0, 1].

### Mapping between Different Omics and Different Technology

For cross-technology mapping across different omics modalities, we produced a shared representation using a two-stage mapping framework based on Canonical Correlation Analysis (CCA)^45^.

In the first stage, SpaOT alignment was performed using only structural information. Intra-modality feature distances were used to characterize the relational structure within each dataset, allowing SpaOT to infer an initial transport plan based purely on structural correspondence to establish soft correspondences between samples across modalities. Based on these correspondences, CCA was applied to project the two modalities into a shared latent representation space. Given molecular profile matrices $X\in R^{n\times d_{1}}$ and $Y\in R^{m\times d_{2}}$ from two modalities, where n, m are the number of units (e.g., cells, spots, or pixels) and $d_{1}$ and $d_{2}$ are the number of the molecular profiles measured in the two modalities, respectively. CCA identifies projection vectors $w_{x}$ and $w_{y}$ by solving:

(20) $$\max_{w_{x},w_{y}}\;corr(Xw_{x},Yw_{y})=\max_{w_{x},w_{y}}\;\frac{w_{x}^{T}\Sigma_{XY}w_{y}}{\sqrt{w_{x}^{T}\Sigma_{XX}w_{x}}\sqrt{w_{y}^{T}\Sigma_{YY}w_{y}}}$$

where $\Sigma_{XX}$ and $\Sigma_{YY}$ denote the within-modality covariance matrices and $\Sigma_{XY}$ denotes the cross-modality covariance matrix. The learned canonical components define a shared latent representation for both modalities.

In the second stage, these shared CCA embeddings were used as molecular profiles in the OT term, while spatial Euclidean distances between samples were used to define the GW term. The final SpaOT alignment was then performed on the resulting mm-spaces constructed from both the shared latent features and spatial structural information.

### Data and preprocessing

Unless otherwise noted, molecular measurements were used as feature vectors (OT term) and pairwise spatial Euclidean distances were used to define the structural components (GW term) of the mm-space.

#### Colorectal cancer (CODEX).

The CRC dataset^26^ contains 140 CODEX images collected from 70 tissue regions across 35 patients, with two images acquired per region and two regions sampled per patient. Patients were categorized into two histopathological groups: Crohn’s-like reaction (CLR; n=17) and diffuse inflammatory infiltration(DII; n=18). Each sample contains measurements for 58 protein markers.

#### Breast cancer (IMC).

The Jackson dataset^31^ comprises 559 IMC images (114 grade 1, 214 grade 2, and 231 grade 3), while the METABRIC dataset^30^ contains 500 images (50 grade 1, 181 grade 2, and 269 grade 3). For disease stratification, grades 1 and 2 were grouped and compared against grade 3. Jackson and METABRIC datasets contained 34 and 37 protein markers, respectively. For inter-dataset stratification, we kept the common 27 protein markers as molecular profiles.

#### Coronal mouse brain.

We analyzed the adult mouse brain spatial transcriptomics dataset consisting of 75 coronal sections generated with Stereo-seq^33^, retaining 40 sections after preprocessing^32^. The dataset contains 17,563 cells and 15,340 genes. SpaOT was applied to adjacent sections to infer inter-section correspondences and reconstruct the three-dimensional tissue architecture. Anatomical regions were subsequently annotated according to the Allen Brain Atlas (ABA)^34^. The reconstructed volumetric data were exported as surface meshes (.obj format) using 3D Slicer without smoothing. The resulting brain structures were visualized in *Plotly*^67^ and colored based on their ABA anatomical annotations.

#### Mouse hypothalamic preoptic area (MERFISH).

The MERFISH dataset comprises 12 coronal sections of the female mouse hypothalamic preoptic area^35^. We reused the preprocessed data from Yu, et al.^32^, which contains 64,373 cells across the 12 sections and 155 gene features per section.

#### Adjacent CRC (Visium-Xenium).

We used paired Visium and Xenium sections from the same donor (10x Genomics P2 CRC dataset)^36^. Visium profiled 18,085 genes across 4,269 spots, whereas Xenium measured 422 genes across 340,837 cells. Xenium cells were down sampled to 10,000 by random sampling, and the 408 genes shared across platforms were retained. Quality control removed low-quality spots/cells and potential doublets using platform-specific thresholds.

#### Quality Control in Adjacent CRC.

Low-quality cells/spots and potential doublets were filtered based on dataset-specific thresholds. For Visium data, spots with fewer than 5 detected genes, more than 350 detected genes, fewer than 300 total counts, or more than 12,000 total counts were removed. For Xenium data, cells with fewer than 5 detected genes, more than 250 detected genes, fewer than 10 total counts, or more than 750 total counts were excluded. The cutoff parameters are determined by manually checking the data distribution (**Supplementary Fig. 4a, 4b**).

#### Mouse cerebellum multi-omics.

The mouse cerebellum dataset^43^ consists of four modalities: spatial transcriptomics, proteomics, metabolomics, and histology images paired with the transcriptomics data. Spatial transcriptomics data were generated using MAGIC-seq, while proteomics data were obtained using the parallel-flow projection and transfer learning across omics data (PLATO) framework. Metabolomics measurements were generated using matrix-assisted laser desorption ionization mass spectrometry imaging (MALDI-MSI). Histology images were spatially paired with the transcriptomics sections.

To evaluate cross-modal spatial correspondence, we selected four representative colocalized gene-protein-metabolite groups according to the original study. The first group, *Mbp*-CLD11-845.68 m/z, was enriched in the Fiber Tracts region. Three additional co-localized groups were analyzed: *Ttr*-CLIC6-811.56 m/z in the Lateral Recess, *mt*-Nd2-GRM1-787.55 m/z in the Molecular Layer, and *Chd9*-FUBP1-909.57 m/z in the Granular Layer. These molecular groups were selected based on their concordant spatial enrichment patterns across transcriptomic, proteomic, and metabolomic modalities and were used to assess the preservation of cross-modal spatial organization.

For mm-space construction in the multi-omics mapping task, in the modalities of spatial transcriptomics, proteomics, and metabolomics, molecular profiles were used as the OT term. For the histology image modality, a pre-trained DINOv3 model^68^ was used to extract pixel-level feature representations as the OT term, and pairwise pixel Euclidean distances were used to define the structural component.

#### Chronic kidney disease (CKD).

We used paired Visium HD and Xenium kidney biopsy sections from a donor with diabetic kidney disease. Visium HD data were available at 8 μm, 16 μm, and segmented single-cell resolutions. Both Visium HD (segmented single-cell resolutions) and Xenium datasets contain expert annotations of glomerular regions with sclerosis severity scores ranging from 0-3, corresponding to healthy, mild, moderate, and severe disease states, respectively. Individual biopsy regions were separated using DBSCAN on spatial coordinates from the four raw biopsy sections. Here, Visium HD provides full-transcriptome measurements at segmented single-cell resolution, whereas Xenium offers *in-situ* single-cell profiling of approximately 300 targeted genes, 295 of which overlap with Visium HD for cross-platform analysis.

#### Label transfer in CKD.

Because the kidney datasets lacked cell-type annotations, labels were transferred from a public scRNA-seq kidney reference dataset containing healthy, acute kidney injury, chronic kidney disease, and diabetes mellitus-resilient samples^69^. Annotation transfer was performed using Seurat's anchor-based label transfer workflow^70^. Briefly, shared features between the reference and query datasets were identified, low-dimensional embeddings were constructed, and anchor-based mapping was used to assign probabilistic cell-type labels to Xenium cells.

### Evaluation criteria

#### Nearest-Neighbor Label Accuracy.

For each cell or spot in slice i, we identified its nearest neighbor in the aligned spatial coordinates of slice i+1. The predicted label was assigned from the nearest neighbor, and accuracy was computed as the proportion of cells whose labels matched between the query cell and its nearest neighbor. We computed a bidirectional score by averaging the forward (i→i+1) and reverse (i+1→i) accuracies weighted by the number of cells in each slice.

#### Centroid Distance.

For each shared cell type, we computed the Euclidean distance between the corresponding spatial centroids in adjacent slices. Distances were averaged across cell types and normalized by the spatial extent of the slice pair to enable comparisons across datasets.

#### Expression cosine similarity.

To evaluate molecular continuity across adjacent slices, we first selected highly variable genes and normalized each cell's expression profile to unit length. For each cell, the cosine similarity between its expression profile and that of its nearest spatial neighbor in the adjacent slice was computed. The final score was obtained as the cell-count-weighted average of forward and reverse nearest-neighbor similarities, with higher values indicating greater transcriptomic consistency across aligned sections.

#### Hull Ratio.

To quantify the spatial compactness of Xenium-to-Visium mappings, we defined a hull ratio metric. For each Xenium cell j, we identified all Visium spots i with transport $p_{ij}>\tau \;(\tau =0.01)$ and computed the convex hull $H_{i}$ of their coordinates. Hull ratio was quantified as

$$HullRatio_{i}=\frac{A(H_{i})}{A(r)}$$

where $A(H_{i})$ denotes the area of the convex hull, A(r) is the area of a Visium spot footprint, and radius r denotes mean nearest-neighbor spot distance. Higher values indicate more spatially dispersed mappings.

#### Cell Conflict.

To quantify the conflict of Visium-to-Xenium alignments, we defined a cell conflict score based on the spatial dispersion of Xenium cells contributing to each Visium spot. For spot i, let $p_{ij}$ denote the transport mass from Xenium cell j with spatial coordinate $s_{j}$. We computed the weighted centroid $\mu_{i}$ and weighted dispersion $S_{i}$:

$$\mu_{i}=\frac{1}{m_{i}}\sum_{j}p_{ij}s_{j},\;\;S_{i}^{2}=\frac{1}{m_{i}}\sum_{j}p_{ij}{|s_{j}-\mu_{i}|}^{2},$$

Where $m_{i}=\sum_{j}p_{ij}$. The cell conflict score was defined as

$$CellConflict_{i}=\frac{S_{i}}{\bar{r}}$$

where $\bar{r}$ is the mean nearest-neighbor distance between Xenium cells. Higher values indicate contributions from spatially distant cells and thus greater mapping conflict.

### Parameter Settings

#### PythonOT.

We used the function *ot.gromov.fused_gromov_wasserstein* with default settings in the analysis. Mm-spaces were constructed as in SpaOT, using molecular profiles as node attributes and pairwise Euclidean distances as the structural component. For graph clustering, cluster centroids were computed using *ot.gromov.fgw_barycenters* with default parameters.

#### moscot.

We used the *AlignmentProblem* module from *moscot.problems.space* in the analysis. Datasets were represented as *AnnData* objects containing molecular features, spatial coordinates, and sample annotations. Molecular features and spatial coordinates were used to define node attributes and geometric relationships, respectively, while sample labels specified source and target domains. Alignments were computed using the default moscot workflow and parameter settings.

#### PASTE2.

We used the *partial_pairwise_align* function with datasets represented as *AnnData* objects containing molecular features, spatial coordinates, and sample annotations. The partial transport parameter s was set to the ground-truth value when available; otherwise, s=1.0 was used. All other parameters were kept at their default or recommended values.

**Logistic Regression (LR), MultiLayer Perceptron (MLP), and Random Forest (RF) classifiers** were implemented using the *scikit-learn* package^64^. For each model, we defined a small parameter grid with commonly used hyperparameter settings and performed lightweight hyperparameter tuning.

For the CRC and intra-dataset breast cancer stratification tasks, the pairwise distance matrices were directly used as model inputs. For the breast cancer translational stratification task, principal component analysis (PCA)^66^ implemented in *scikit-learn* was first applied for dimensionality reduction prior to classification. After component selection, 35 principal components were retained when using METABRIC to predict Jackson, whereas 60 principal components were retained for the reverse prediction task. To confirm that the SpaOT pairwise distance matrices were not sensitive to the choice of PCA dimensionality and to rule out potential bias from selecting a specific number of principal components, we evaluated performance across a range of 10-80 principal components. The results demonstrated consistent performance across this range for both prediction tasks (**Supplementary Fig. 8a**, METABRIC to predict Jackson; **Supplementary Fig. 8b**, Jackson to predict METABRIC).

### Functional Analysis

**Differential expression analysis** between SPP1-enriched and SPP1-depleted samples was performed using the *rank_genes_groups* function in Scanpy^38^ with the Wilcoxon rank-sum test^71^. Genes with adjusted *P* < 0.05 and ∣logFC∣>0.3 were considered differentially expressed.

**Pathway enrichment analysis** was performed using *ReactomePA*^39^ and **Gene Ontology enrichment analysis**^40^ was performed using *clusterProfiler* on significant DEGs (*adjusted P* < 0.05) converted to Entrez Gene IDs. Significantly enriched pathways were identified using a hypergeometric test with Benjamini–Hochberg correction.

### Cell-cell Communication Analysis

**CellChat**^49^ was applied to infer ligand-receptor-mediated cell-cell communication networks using the “*TriMean*” function. All analyses were performed following the standardized workflow recommended in the official CellChat tutorial. Subsequently, significant ligand-receptor interaction pairs were extracted and compared between the healthy and disease groups. Venn diagram analysis was then performed to identify shared and condition-specific ligand-receptor interactions between the two groups.

**COMMOT**^47^ was applied following the official tutorial and recommended parameter settings. Briefly, ligand-receptor pairs were first filtered to retain biologically relevant interactions supported by the data. COMMOT was then used to infer pathway-level spatial communication probabilities by integrating molecular expression profiles with spatial proximity information. Finally, pathway-specific communication vector fields were generated to visualize the inferred directions and spatial patterns of cell-cell signaling across the tissue.

## Supplementary Material

This is a list of supplementary files associated with this preprint. Click to download.

- SupplementaryNotes.pdf
- SupplementaryFig.pdf

## Data Availability

All relevant data supporting the key findings of this study are available within the article and its supplementary information files. The human CRC CODEX dataset used in the colorectal cancer case study is available at https://data.mendeley.com/datasets/mpjzbtfgfr/1. The IMC breast cancer from Jackson et al is available at https://doi.org/10.5281/zenodo.3518284, from METABRIC is available at https://idr.openmicroscopy.org/ under accession code idr0076. The adjacent Coronal Mouse Brain dataset is available at https://www.10xgenomics.com/datasets/mouse-brain-coronal-section-1-ffpe-2-standard, and adjacent MERFISH Mouse Hypothalamic Preoptic dataset is available at https://datadryad.org/dataset/doi:10.5061/dryad.8t8s248. The 10x Genomics CRC dataset is available at https://www.10xgenomics.com/platforms/visium/product-family/dataset-human-crc. The muti-omics Mouse Cerebellum dataset is available at https://github.com/bioinfobiols/Flow2Spatial/tree/main/datasets. The HuBMAP reference or disease tissue data can be access through the HuBMAP publication page upon acceptance of the peer-reviewed manuscript. Source data are provided with this paper.

## CODE AVAILABILITY

The source code of SpaOT is freely available at https://github.com/ShuangWang1/SpaOT. The specific version of the code and simulation data associated with this publication is accessible at https://github.com/ShuangWang1/SpaOT_reproducibility.
